# A 42-Markers Pharmacokinetic Study Reveals Interactions of Berberine and Glycyrrhizic Acid in the Anti-diabetic Chinese Medicine Formula Gegen-Qinlian Decoction

**DOI:** 10.3389/fphar.2018.00622

**Published:** 2018-06-19

**Authors:** Xue Qiao, Qi Wang, Shuang Wang, Yi Kuang, Kai Li, Wei Song, Min Ye

**Affiliations:** State Key Laboratory of Natural and Biomimetic Drugs, School of Pharmaceutical Sciences, Peking University, Beijing, China

**Keywords:** bioavailability, synergistic action, gegen-qinlian decoction, berberine, glycyrrhizic acid

## Abstract

Herbal medicines are commonly used as compound formulas in clinical practice to achieve optimal therapeutic effects. However, the combination mechanisms usually lack solid evidence. In this study, we report synergistic interactions through altering pharmacokinetics in Gegen-Qinlian Decoction (GQD), an anti-diabetic Chinese medicine formula. A multi-component pharmacokinetic study of GQD and the single herbs was conducted by simultaneously monitoring 42 major bioactive compounds (markers) in rats plasma using LC/MS/MS within 30 min. GQD could remarkably improve the plasma concentrations of berberine (BER) and other alkaloids in Huang-Lian by at least 30%, and glycyrrhizic acid (GLY) from Gan-Cao played a major role. A Caco-2 cell monolayer test indicated that GLY improved the permeability of BER by inhibiting P-glycoprotein. Although GLY alone did not show observable effects, the co-administration of GLY (ig, 50 or 80 mg/kg) could improve the anti-diabetic effects of berberine (ig, 50 mg/kg) in db/db mice in a dose-dependent manner. The blood glucose decreased by 46.9%, whereas the insulin level increased by 40.8% compared to the control group. This is one of the most systematic studies on the pharmacokinetics of Chinese medicine formulas, and the results demonstrate the significance of pharmacokinetic study in elucidating the combination mechanisms of compound formulas.

## Introduction

Herbal medicines are usually used in the form of compound formulas in clinical practice (Wang et al., 2011; Li et al., 2013). It is generally believed that the combination of different herbs could increase therapeutic effects or alleviate toxicity (Wang et al., 2008). Traditional Chinese Medicine (TCM) has a sophisticated formulation theory, where the component herbs belong to four classes, *Jun, Chen, Zuo*, and *Shi*, according to their roles in the formula (Che et al., 2013). Although this theory has been used for at least 2,000 years in clinical practice, solid scientific evidence to interpret the combination mechanisms for most formulas is still lacking.

A number of pharmacological studies have demonstrated the combination of different herbs in a TCM formula could lead to synergistic effects, though the compounds responsible for the interactions and the related mechanisms are not clear (Li et al., 2010). Many clinical drug-drug or herb-drug interactions are due to alterations in pharmacokinetics (PK) (Windrum et al., 2000; König et al., 2013). Pharmacokinetic study may be able to unveil TCM formulation mechanisms. Nonetheless, PK study of TCM formulas has been a big challenge. First, TCM formulas may contain hundreds of compounds, and the bioactive compounds are usually unknown, which renders it difficult to choose appropriate markers for PK study. Second, it is challenging to simultaneously determine a big array of compounds in biofluids with different structural types at different concentration levels. Therefore, most PK studies of TCM formulas only monitor several major compounds (Wang et al., 2009; Munekage et al., 2013). Although a few studies determined more markers, like 23 compounds in a herb couple, pharmacokinetics of the formula, and the components herbs were not compared, and the interactions could not be revealed (Zhou et al., 2015).

Gegen-Qinlian Decoction (GQD) is a well-known TCM formula derived from the classical book *Shang Han Lun* (Treatise on Febrile Diseases) in Han Dynasty of Chinese history (202 BC-220 AD). Today, GQD and related modern patent drugs (such as GQD tablets or pills) are widely used to treat type 2 diabetes, diarrhea, and influenza in clinical practice (Xu et al., 2015). GQD is composed of four herbs, namely Puerariae Lobatae Radix (Ge-Gen in Chinese, GG), Scutellariae Radix (Huang-Qin, HQ), Coptidis Rhizoma (Huang-Lian, HL), and Glycyrrhizae Radix et Rhizoma (Gan-Cao, GC). We have investigated chemical constituents and pharmacokinetics of these single herbs in the past years (Qiao et al., 2012; Ji et al., 2015, 2016). Recently, we have also analyzed the chemical composition of GQD by 2DLC/MS, and identified at least 280 compounds, including flavonoid glycosides, saponins, alkaloids, and different types of free phenolic compounds (Qiao et al., 2016a). Furthermore, the contents of 50 major compounds were quantitatively determined by LC/MS/MS (Wang et al., 2016). Based on the above studies, GQD has become one of the few TCM formulas whose chemical composition is systematically clarified. To further dissect the effective components and mechanisms of action, we have also studied the metabolic pathways of GQD after oral administration in rats, and identified at least 131 metabolites. These metabolites are derived from 46 bioactive parent compounds (Qiao et al., 2016b). However, few reports are available on the pharmacokinetics of GQD. A recent report monitored the pharmacokinetics of 12 compounds, though interactions between the component herbs or compounds were not involved (Zhang et al., 2015).

In this work, we conducted a comprehensive pharmacokinetic study of GQD in rats by simultaneously monitoring 42 major bioactive compounds within 30 min using liquid chromatography coupled with tandem mass spectrometry (LC/MS/MS). The impact of herbal combinations on pharmacokinetics was discussed by comparing the PK parameters of GQD and the single herbs. Furthermore, we discovered the synergistic effects of berberine and glycyrrhizic acid to enhance the anti-diabetic effects of GQD, and the mechanism on regulating P-glycoprotein was revealed.

## Materials and methods

### Chemicals and reagents

Compounds **1**–**7**, **37**, **39**, **41** were from Ge-Gen; **8**–**18**, **38**, **42** were from Huang-Qin; **26**–**36** and **40** were isolated from Gan-Cao. They were purified by the authors, and the structures were characterized by UV, MS, ^1^H and ^13^C NMR spectroscopic analyses. Compounds **19**, **21**–**25** were purchased from Mansite Biotechnology Co., Ltd. Compound **20** was from Feiyu Fine Chemical. The internal standard butein 4-*O*-β-D-glucoside was isolated from *Sophora alopecuroides* L. by the authors. Their purities were >98% by HPLC/UV analysis. Ultra-pure water was prepared with a Milli-Q water purification system. Heparin was purchased from Beijing Solarbio Science & Technology Co., Ltd. Other reagents were of analytical grade. Reagents for the Caco-2 model were described in Supplemental Data.

### Herbal materials and extracts

GQD water decoction was prepared according to its original record in *Shang-Han-Lun* (Qiao et al., 2016a, see Supplemental Data). Briefly, Ge-Gen, Huang-Qin, Huang-Lian, and Gan-Cao were extracted in the ratio of 8:3:3:2 (25.04, 9.43, 9.43, and 6.31 g, respectively). GQD_−GC_ and single herb extracts was prepared using the same procedure. For GQD_−GC_, Gan-Cao was removed from GQD. Final concentrations of the extracts were 1.0 g/mL for GQD, GQD_−GC_, Ge-Gen; and 0.5 g/mL for Huang-Qin, Huang-Lian and Gan-Cao. Contents of marker compounds in GQD were from our previous report (Wang et al., 2016), except for three compounds (**35**, **41**, **42**) which were determined in this study.

### Pharmacokinetic study

Male Sprague-Dawley rats (220–250 g) were used for PK study, and the details were described in Supplemental Data. All procedures were in accordance with the National Institutes of Health *Guide for the Care and Use of Laboratory Animals*.

For PK study of GQD and its component herbs, rats were randomly divided into six groups (*n* = 10 for each group) before oral administration. GQD, GQD_−GC_, Ge-Gen, Huang-Qin, Huang-Lian, and Gan-Cao extracts were administrated at the doses of 18.9, 16.5, 9.45, 3.54, 3.54, and 2.36 g/kg, respectively. The dosage of GQD is equivalent to a 1.5-fold clinical dosage (12.5 g/kg, Qiao et al., 2016b), and the dosage of component herbs were equivalent to their contents in the formula. Retro-orbital blood samples (400 μL) were collected into heparinized tubes at 0.08, 0.25, 0.42, 0.58, 1, 2, 4, 8, 12, 24, 36, and 48 h after treatment.

For the PK study of berberine and glycyrrhizic acid, rats were randomly divided into four groups (*n* = 8 for each group) before oral administration. Two groups were respectively treated with 59.4 mg/kg of berberine and 84.8 mg/kg of glycyrrhizic acid; the third group was treated with 59.4 mg/kg of berberine and 2.36 g/kg of licorice water decoction (0.5 g/mL); and the last group received 59.4 mg/kg of berberine and 84.8 mg/kg of glycyrrhizic acid. The dosage of single compounds were calculated according to their contents in the administrated GQD extract. Sample collection were the same as described for GQD.

All blood samples were immediately centrifuged at 6,000 rpm (4°C) for 20 min to obtain plasma. The plasma samples were stored at −80°C. Samples were prepared for LC/MS/MS analysis as described in our previous study (Qiao et al., 2012), as described in Supplemental Data.

### Caco-2 cell monolayer permeability test

Caco-2 cells were purchased at passage 18 and all experiments were performed during passages 24–35 following our previously reported method (Wang et al., 2015), as described in Supplemental Data. The final concentration of GQD, GQD_−GC_, and Huang-Lian water extracts were 32, 28, and 6 μg/mL, respectively. The concentrations were set to match the ratio of component herbs (GG:HQ:HL:GC = 8:3:3:2). The final concentration of berberine was 5 μM. Verapamil and rifampicin were used as P-gp inhibitor and inducer of Caco-2 cells, respectively.

### LC/MS/MS analysis

A Finnigan TSQ Quantum triple quadrupole mass spectrometer was connected to a Surveyor HPLC via ESI interface (ThermoFisher). High purity nitrogen was used as the sheath and auxiliary gas; Ultra-high purity argon was used as the collision gas (1.5 mTorr). Q1 and Q3 quadrupoles were set at unit resolution. Samples were separated on a Waters XTerra MS-C_18_ column (2.1 × 150 mm, 3.5 μm) protected with an XTerra MS-C_18_ guard column (3.9 × 20 mm, 5 μm). The column temperature was 40°C. The mobile phase consisted of acetonitrile containing 2% (*v*/*v*) methanol (A) and water containing 0.1% (*v*/*v*) formic acid (B). A linear gradient elution program was used as follows: 0 min, 5% A; 8 min, 25% A; 12 min, 25% A; 15 min, 40% A; 23 min, 80% A; 25 min, 95% A; 30 min, 95% A. The flow rate was 0.2 mL/min. An aliquot of 5 μL was injected for analysis. UV spectra were obtained by scanning from 200 to 400 nm. The mass spectrometer was operated in the (+)-ESI mode. The optimized parameters were as follows: sheath gas (N_2_), 50 arb; auxiliary gas (N_2_), 5 arb; spray voltage, 4.0 kV; capillary temperature, 330°C; collision energy, see Table S1. Analytes were detected in the selected reaction monitoring (SRM) mode. Data were processed by Xcalibur 2.0.7 software (ThermoFisher).

### Evaluation of anti-diabetic effects in Db/Db mice

Eight-week-old male db/db mice (30–35 g) were used, and the details were described in Supplemental Data. The mice were randomly divided into eight groups. Mice were orally administered with metformin (100 mg/kg/d, *n* = 6), berberine (50 mg/kg/d, *n* = 6), glycyrrhizic acid (80 mg/kg/d, *n* = 6), berberine (50 mg/kg/d) combined with low-dose of glycyrrhizic acid (50 mg/kg/d, *n* = 6), berberine (50 mg/kg/d) combined with high-dose of glycyrrhizic acid (80 mg/kg/d, *n* = 6), berberine (50 mg/kg/d) combined with Gan-Cao (2.2 g/kg/d, *n* = 7), and GQD (18.2 g/kg/d, *n* = 7) for 4 weeks. For the control group, the rats were administered with an equal volume of water. On day 28, after fasted for 12 h, retro-orbital blood samples were collected, stand for 1 h, and then centrifuged (6,000 rpm, 4°C) for 20 min to obtain serum. Biochemical indicators such as blood glucose and insulin were determined on a Mindray BS-800 analyzer (Mindray Bio-Medical Electronics Co.).

### Data analysis

LC/MS/MS calibration and quantitation data were processed with Xcalibur 2.0.7 software (ThermoFisher). The maximal plasma concentrations (*C*_*max*_) and their time of occurrence (*T*_max_) were obtained directly from the measured data. Other pharmacokinetic parameters were calculated by non-compartment or compartment models using WinNonlin® software (v6.1, Pharsight). Unless otherwise stated, results were expressed as the mean ± SD. Statistical significance in differences of the means was determined by Student's *t*–test, with a significance level of *P* < 0.05.

## Results

### Multi-component pharmacokinetics of GQD

**Figure 1:**
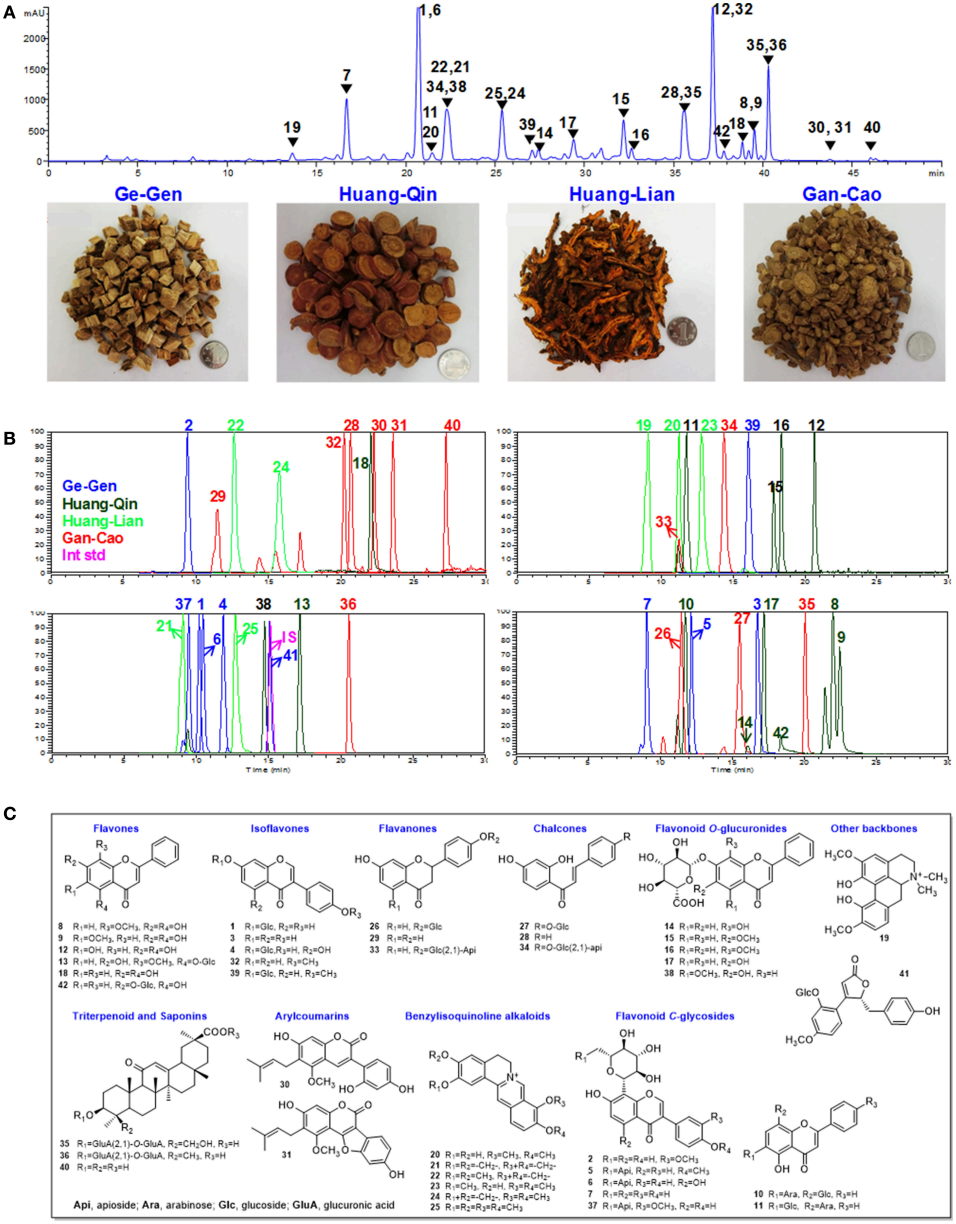
Chemical structures and chromatograms of GQD. **(A)** HPLC-UV chromatogram for Gegen-Qinlian Decoction (GQD) at 270 nm, and pictures of the four component herbs. **(B)** LC/MS/MS chromatograms for the 42 analytes and the internal standard (IS, butein 4-*O*-glucoside) in rats plasma. **(C)** Chemical structures of **1**-**42**. Daidzin (**1**), 3′-methoxypuerarin (**2**), daidzein (**3**), genistin (**4**), formononetin 8-*C*-apiofuranosyl(1,6)glucoside (**5**), genistein 8-*C*-apiofuranosyl(1,6)glucoside (**6**), puerarin (**7**), wogonin (**8**), oroxylin A (**9**), chrysin 6-*C*-α-L-arabinoside-8-*C*-β-D-glucoside (**10**), chrysin 6-*C*-β-D-glucoside-8-*C*-β-L-arabinoside (**11**), baicalein (**12**), wogonin 5-*O*-glucoside (**13**), norwogonin 7-*O*-glucuronide (**14**), oroxylin A 7-*O*-glucuronide (**15**), wogonoside (**16**), baicalin (**17**), chrysin (**18**), magnoflorine (**19**), demethyleneberberine (**20**), coptisine (**21**), epiberberine (**22**), jatrorrhizine (**23**), berberine (**24**), and palmatine (**25**), liquiritin (**26**), isoliquiritin (**27**), isoliquiritigenin (**28**), liquiritigenin (**29**), glycycoumarin (**30**), glycyrol (**31**), formononetin (**32**), liquiritin apioside (**33**), isoliquiritin apioside (**34**), licorice saponin G2 (**35**), glycyrrhizic acid (**36**), 3'-methoxymirificin (**37**), lateriflorein 7-*O*-glucuronide (**38**), ononin (**39**), glycyrrhetinic acid (**40**), (4*S*)-puerol B 2″-*O*-glucopyranoside (**41**), chrysin 7-*O*-glucuronide (**42**).

Our previous study suggested that 46 compounds could expose in plasma or urine after oral administration of GQD (Qiao et al., 2016b). In this work, we conducted a pharmacokinetic study of GQD to simultaneously monitor 42 marker compounds. They represent the major peaks in the HPLC chromatogram of GQD, and cover majority of the 46 exposable compounds, except for a few minor ones such as 4′-methoxypuerarin and naringenin. Among them, 10, 13, 7, and 12 compounds were from Ge-Gen, Huang-Qin, Huang-Lian and Gan-Cao, respectively. Their structures are shown in Figure 1. Most of these compounds possess significant bioactivities, including anti-inflammatory, antioxidant, antiviral, and anti-diabetic activities (Li et al., 2011; Zhou et al., 2014; Ji et al., 2015, 2016; Ma et al., 2016).

To determine these compounds, a 30-min LC/MS/MS method in the SRM mode method was established and optimized (Figure 1). The method was fully validated to comply with the FDA guidance for bioanalysis, including linearity, intra-day and inter-day variation, recovery, matrix effect, and analyte stability (Tables S2–S5).

After oral administration of GQD (1.5-fold of clinical dosage), four types of compounds showed high plasma exposure: flavonoid *O*-glucuronides from Huang-Qin such as **14**, **15**, **16**, **17**, **38**, and **42** (*AUC*_last_>700 h·ng/mL); saponins from Gan-Cao such as glycyrrhizic acid (**36**) (*AUC*_last_ = 870.76 ± 570.92 h·ng/mL); flavonoid *C*- and *O*- glycosides from Ge-Gen and Gan-Cao including **1**, **2**, **7**, **26**, **33**, and **39** (*AUC*_last_ >300 h·ng/mL); and alkaloids from Huang-Lian such as **19**, **21**, **23**, and **24** (*AUC*_last_ >100 h·ng/mL). PK behaviors for different types of compounds varied remarkably. Isoquinoline alkaloids, flavonoid *O*- and *C*-glycosides, flavonoid *O*-glucuronides, and saponins reached the maximal plasma concentrations sequentially.

### Herbal combinations remarkably altered the pharmacokinetics of GQD compounds

**Figure 2:**
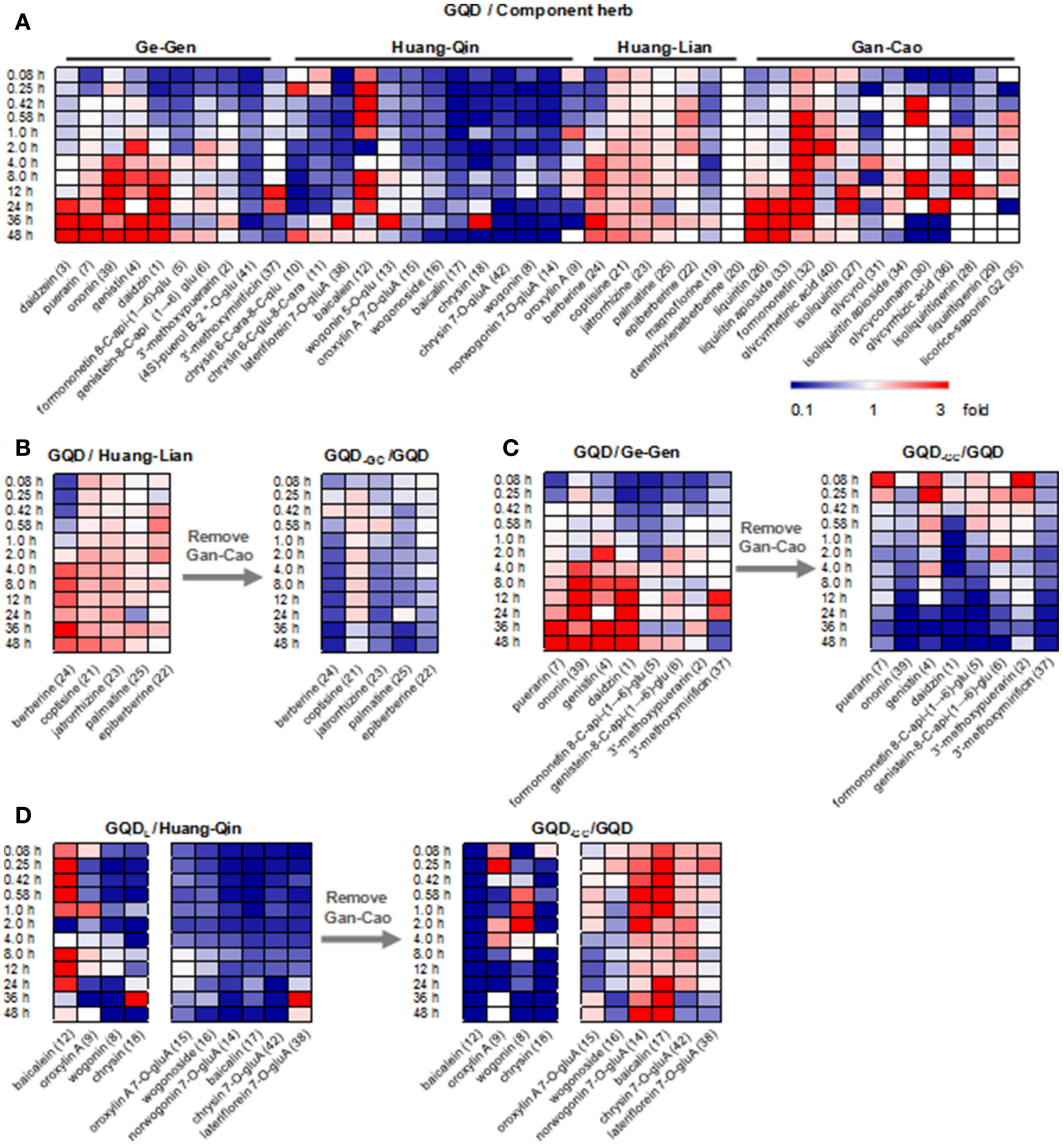
Comparison of plasma concentrations to reveal PK interactions. **(A)** Heatmap of GQD vs. component herb groups. **(B)** Heatmap of GQD vs. GQD_−GC_ for alkaloids in Huang-Lian. **(C)** Heatmap of GQD vs. GQD_−GC_ for glycosides in Ge-Gen. **(D)** Heatmap of GQD vs. GQD_−GC_ for phenols and flavonoid *O*-glucuronides in Huang-Qin. Two groups of average plasma concentrations (*n* = 10) were compared in each heatmap. They were referred to as “X/Y”, where Y is the reference group. GQD-GC represents the Gegen-Qinlian Decoction without Gan-Cao.

By comparing the PK parameters of the 42 compounds between the GQD group and the single herb groups, we found that herbal combinations remarkably changed the PK behaviors of many GQD compounds (Figure 2).

**Figure 3:**
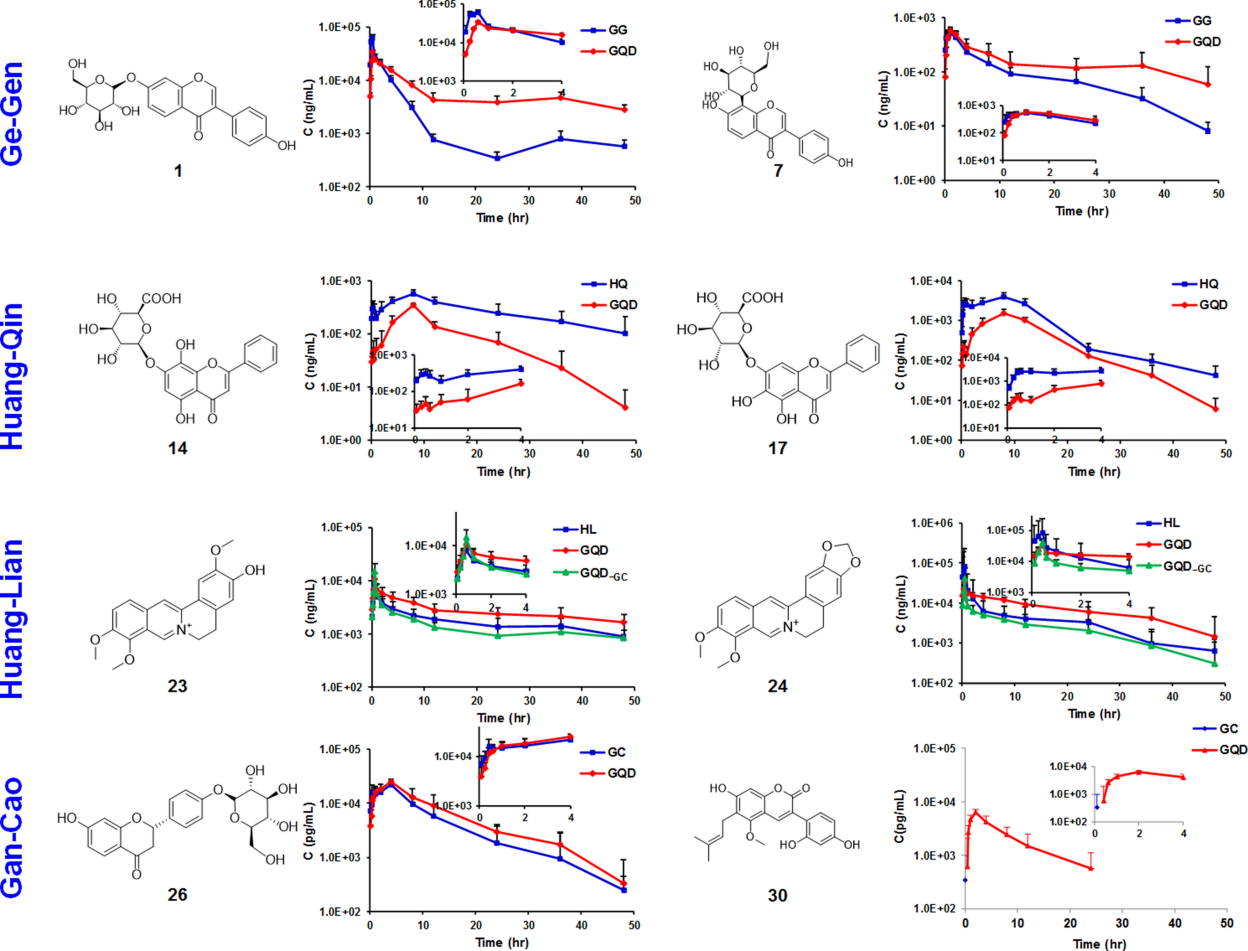
Time-plasma concentration curves of selected marker compounds in GQD and in component herb groups. Ge-Gen: daidzin (**1**), puerarin (**7**). Huang-Qin: norwogonin 7-*O*-glucuronide (**14**), baicalin (**17**). Huang-Lian: jatrorrhizine (**23**), berberine (**24**). Gan-Cao: liquiritin (**26**), glycycoumarin (**30**).

GQD could improve the bioavailability of benzylisoquinoline alkaloids from Huang-Lian. Particularly, the *AUC*_last_ values of **21**, **23**, **24**, and **25** increased by 49.1, 63.6, 96.5, and 32.7%, respectively, when compared with the Huang-Lian group. For instance, the *AUC*_last_ value of **23** and **24** were 194.57 ± 25.67 and 389.12 ± 187.34 h·ng/mL in the GQD group, and 118.93 ± 52.37 and 197.98 ± 76.94 h·ng/mL in the Huang-Lian group, respectively. Interestingly, the *AUC*_last_ of **23** and **24** decreased to 105.04 ± 34.20 and 148.97 ± 70.83 h·ng/mL in the GQD_−GC_ group (Figure 3). These results indicated that Gan-Cao played a critical role in improving the bioavailabilities of Huang-Lian alkaloids such as berberine (**24**) and jatrorrhizine (**23**).

For the flavonoid *O*- and *C*-glycosides from Ge-Gen (**1**, **4**, **5**, **7**, and **39**), their bioavailabilities were higher in the GQD group than in the Ge-Gen group. For instance, *AUC*_last_ for daidzin (**1**) and puerarin (**7**) was 345.71 ± 30.34 and 8611.51 ± 765.80 h·ng/mL in the GQD group, and 290.46 ± 42.22 and 5021.69 ± 535.79 h·ng/mL in the Ge-Gen group, respectively (Table 1 and Figure 3).

**Table 1:** The pharmacokinetic parameters for 42 analytes in different groups.

**Groups**	**Analytes**	***T*_max_ (h)**	***C*_max_ (ng/mL)**	*AUC*_last_ (h*ng/mL)	***T*_1/2_ (h)**
GQD	**1**	0.88 ± 0.11	27.35 ± 3.34	345.71 ± 30.34	3.96 ± 1.02
GQD-GC		0.58 ± 0.24	52.38 ± 2.21	281.76 ± 61.63	1.63 ± 0.14
GG		0.38 ± 0.04	57.97 ± 0.77	290.46 ± 42.22	0.74 ± 0.33
GQD	**2**	1.62 ± 0.22	206.44 ± 12.98	5150.57 ± 441.22	7.59 ± 4.66
GQD-GC		0.86 ± 0.73	188.54 ± 9.78	2993.39 ± 719.23	4.89 ± 0.73
GG		0.83 ± 0.35	183.43 ± 24.32	4426.7 ± 597.48	5.47 ± 2.71
GQD	**3**	8.00 ± 12.83	186.03 ± 36.23	3051.89 ± 998.38	6.23 ± 1.36
GQD-GC		0.25 ± 0.32	83.46 ± 3.23	674.13 ± 603.86	4.28 ± 1.47
GG		8.00 ± 0.14	187.81 ± 10.38	2644.89 ± 1146.74	5.12 ± 0.87
GQD	**4**	1.04 ± 0.78	4.97 ± 1.11	72.56 ± 15.23	6.93 ± 0.73
GQD-GC		1.04 ± 0.75	8.08 ± 3.19	88.19 ± 24.32	8.17 ± 2.43
GG		1.00 ± 2.31	3.09 ± 1.99	51.68 ± 24.17	6.88 ± 5.23
GQD	**5**	0.76 ± 3.76	16.01 ± 5.44	168.12 ± 83.27	7.03 ± 2.31
GQD-GC		0.52 ± 0.65	6.87 ± 2.05	26.04 ± 7.29	5.23 ± 1.35
GG		0.39 ± 0.17	18.35 ± 5.10	85.01 ± 19.22	8.98 ± 2.34
GQD	**6**	8.39 ± 0.23	11.27 ± 3.32	98.8 ± 24.76	6.77 ± 3.44
GQD-GC		1.56 ± 1.53	12.07 ± 7.87	96.28 ± 105.30	2.15 ± 1.70
GG		0.73 ± 0.3	11.27 ± 1.8	167.5 ± 33.61	5.57 ± 3.48
GQD	**7**	1.20 ± 1.49	577.23 ± 56.32	8611.51 ± 765.80	5.58 ± 2.90
GQD-GC		0.52 ± 0.07	415.99 ± 231.78	5612.93 ± 278.74	8.29 ± 2.22
GG		0.59 ± 0.01	487.85 ± 23.87	5021.69 ± 535.79	6.36 ± 4.39
GQD	**8**	7.89 ± 0.99	12.87 ± 7.39	282.08 ± 11.34	6.63 ± 2.38
GQD-GC		8.00 ± 2.12	10.21 ± 6.81	156.38 ± 73.4	5.64 ± 1.09
HQ		8.02 ± 2.48	38.04 ± 7.22	473.14 ± 34.29	5.45 ± 1.22
GQD	**9**	*n*.*a*.	*n*.*a*.	*n*.*a*.	*n*.*a*.
GQD-GC		*n*.*a*.	*n*.*a*.	*n*.*a*.	*n*.*a*.
HQ		0.08 ± 0.53	43.56 ± 3.04	351.38 ± 173.54	5.29 ± 2.33
GQD	**10**	2.34 ± 0.08	11.09 ± 2.84	155.13 ± 13.94	1.62 ± 0.98
GQD-GC		5.40 ± 0.96	8.46 ± 2.53	53.86 ± 30.15	3.57 ± 1.88
HQ		5.41 ± 0.76	28.64 ± 14.33	412.9 ± 270.83	3.67 ± 1.09
GQD	**11**	4.53 ± 0.19	23.2 ± 1.04	229.1 ± 40.33	3.07 ± 1.48
GQD-GC		3.83 ± 0.14	31.62 ± 5.04	151.46 ± 90.43	2.51 ± 1.21
HQ		4.49 ± 3.42	66.42 ± 9.85	1203.45 ± 263.96	2.90 ± 1.76
GQD	**12**	0.42 ± 0.03	11.11 ± 0.66	108.29 ± 46.96	7.57 ± 0.21
GQD-GC		*n*.*a*.	*n*.*a*.	*n*.*a*.	*n*.*a*.
HQ		1.00 ± 0.64	3.46 ± 0.34	19.48 ± 11.23	5.99 ± 1.23
GQD	**13**	2.00 ± 1.29	1.83 ± 0.14	52.99 ± 7.56	16.13 ± 4.22
GQD-GC		2.00 ± 4.23	5.17 ± 0.26	28.16 ± 3.04	13.11 ± 3.96
HQ		0.10 ± 0.07	1.92 ± 0.13	42.75 ± 6.01	16.08 ± 5.45
GQD	**14**	7.05 ± 1.23	379.34 ± 98.54	5267.25 ± 987.31	4.88 ± 2.87
GQD-GC		6.17 ± 8.34	359.48 ± 34.34	6033.79 ± 272.31	4.28 ± 1.63
HQ		7.36 ± 0.08	547.17 ± 73.77	10951.14 ± 2530.70	5.10 ± 0.33
GQD	**15**	8.05 ± 1.29	174.96 ± 29.58	6123.69 ± 477.76	8.84 ± 1.40
GQD-GC		8.50 ± 0.12	77.97 ± 34.16	3573.21 ± 1034.39	6.24 ± 2.21
HQ		8.41 ± 0.6	153.45 ± 89.19	7635.05 ± 665.2	8.27 ± 3.40
GQD	**16**	7.05 ± 1.27	213.32 ± 39.28	10342.34 ± 112.34	8.13 ± 2.98
GQD-GC		7.37 ± 4.3	98.03 ± 32.33	18558.81 ± 2707.6	5.30 ± 0.27
HQ		2.00 ± 0.26	169.22 ± 34.29	21491.67 ± 9287.63	8.77 ± 3.15
GQD	**17**	7.29 ± 1.38	1183.39 ± 870.40	22274.24 ± 11236.50	4.80 ± 3.27
GQD-GC		7.71 ± 1.34	1888.05 ± 988.32	39552.49 ± 2609.08	5.34 ± 2.55
HQ		7.79 ± 0.99	4043.24 ± 584.50	52619.53 ± 35547.30	3.32 ± 1.08
GQD	**18**	*n*.*a*.	*n*.*a*.	*n*.*a*.	*n*.*a*.
GQD-GC		*n*.*a*.	*n*.*a*.	*n*.*a*.	*n*.*a*.
HQ		0.58 ± 0.23	4.1 ± 0.44	41.28 ± 1.34	4.87 ± 1.43
GQD	**19**	4.13 ± 1.21	19.71 ± 5.67	198.48 ± 74.90	8.35 ± 1.26
GQD-GC		0.28 ± 0.13	5.21 ± 1.20	47.34 ± 9.31	4.54 ± 1.43
HL		4.21 ± 3.47	76.41 ± 23.45	556.12 ± 223.98	10.21 ± 4.34
GQD	**20**	*n*.*a*.	*n*.*a*.	*n*.*a*.	*n*.*a*.
GQD-GC		*n*.*a*.	*n*.*a*.	*n*.*a*.	*n*.*a*.
HL		*n*.*a*.	*n*.*a*.	*n*.*a*.	*n*.*a*.
GQD	**21**	0.74 ± 0.32	6.56 ± 4.03	109.2 ± 73.23	5.20 ± 1.37
GQD-GC		0.69 ± 0.76	8.39 ± 1.3	167.46 ± 24.35	3.87 ± 0.27
HL		0.68 ± 0.65	5.33 ± 1.35	73.23 ± 21.23	3.83 ± 1.34
GQD	**22**	0.58 ± 0.12	7.21 ± 3.62	29.35 ± 21.32	4.84 ± 0.13
GQD-GC		0.54 ± 0.17	9.35 ± 1.71	26.41 ± 11.02	4.97 ± 0.85
HL		0.58 ± 0.23	3.53 ± 0.34	16.73 ± 9.45	4.34 ± 0.18
GQD	**23**	0.72 ± 0.11	8.54 ± 0.05	194.57 ± 25.67	6.97 ± 1.77
GQD-GC		0.63 ± 0.32	9.14 ± 0.82	105.04 ± 34.2	3.12 ± 0.13
HL		0.61 ± 0.11	6.6 ± 0.59	118.93 ± 52.37	5.15 ± 1.74
GQD	**24**	0.39 ± 0.76	28.20 ± 17.45	389.12 ± 187.34	11.42 ± 2.78
GQD-GC		0.35 ± 0.23	32.28 ± 13.20	148.97 ± 70.83	7.09 ± 0.65
HL		0.29 ± 1.12	76.65 ± 27.89	197.98 ± 76.94	9.30 ± 1.67
GQD	**25**	0.17 ± 0.03	17.15 ± 2.98	68.34 ± 39.23	1.62 ± 1.82
GQD-GC		0.26 ± 0.15	4.75 ± 10.23	43.4 ± 16.99	3.03 ± 2.36
HL		0.25 ± 1.25	5.96 ± 1.35	51.5 ± 27.8	3.25 ± 2.43
GQD	**26**	2.50 ± 4.76	20.76 ± 1.86	416.94 ± 210.31	12.05 ± 1.16
GC		1.34 ± 3	18.62 ± 7.53	237.25 ± 92.4	7.84 ± 0.38
GQD	**27**	0.49 ± 1.01	2.59 ± 2.43	11.93 ± 2.4	1.62 ± 0.33
GC		0.40 ± 1.23	2.17 ± 0.98	8.46 ± 2.99	0.91 ± 0.08
GQD	**28**	5.33 ± 0.89	2.81 ± 0.87	39.2 ± 8.9	3.55 ± 0.08
GC		0.41 ± 0.47	1.16 ± 0.52	1.28 ± 0.03	0.28 ± 0.08
GQD	**29**	1.00 ± 0.93	27.35 ± 9.97	153 ± 9.34	11.23 ± 7.69
GC		1.00 ± 2.34	22.63 ± 0.22	75.36 ± 81.09	9.84 ± 3.52
GQD	**30**	2.00 ± 1.24	7.47 ± 8.1	37.91 ± 29.93	11.35 ± 3.38
GC		*n*.*a*.	*n*.*a*.	*n*.*a*.	*n*.*a*.
GQD	**31**	*n*.*a*.	*n*.*a*.	*n*.*a*.	*n*.*a*.
GC		*n*.*a*.	*n*.*a*.	*n*.*a*.	*n*.*a*.
GQD	**32**	2.92 ± 0.08	8.62 ± 0.39	94.18 ± 32.63	23.78 ± 16.76
GC		0.33 ± 0.13	1.71 ± 1	19.86 ± 7.99	3.22 ± 0.87
GQD	**33**	2.09 ± 0.24	12.6 ± 0.14	514.13 ± 7.53	15.43 ± 2.23
GC		1.59 ± 0.09	13.53 ± 0.78	278.49 ± 1.41	13.11 ± 6.66
GQD	**34**	3.68 ± 8.09	3.28 ± 1.23	40.83 ± 10.23	5.35 ± 1.41
GC		2.89 ± 0.76	2.67 ± 1.33	32.49 ± 5.6	6.05 ± 0.89
GQD	**35**	3.89 ± 1.41	10.3 ± 6.38	102.23 ± 53.34	2.53 ± 0.14
GC		3.70 ± 1.41	8.98 ± 2.49	81.01 ± 27.7	2.30 ± 6.79
GQD	**36**	6.90 ± 0.28	55.62 ± 14.34	870.76 ± 570.92	4.78 ± 9.98
GC		5.89 ± 0.98	46.44 ± 0.89	790.47 ± 362.54	4.08 ± 1.07
GQD	**37**	0.36 ± 0.03	9.94 ± 3.68	86.26 ± 23.9	1.04 ± 0.04
GQD-GC		0.35 ± 0.42	7.22 ± 3.27	42.45 ± 11.28	1.62 ± 0.39
GG		0.36 ± 0.74	11.55 ± 0.56	47.4 ± 28.74	1.37 ± 0.19
GQD	**38**	0.44 ± 3.87	48.75 ± 3.88	699.68 ± 77.05	4.33 ± 3.23
GQD-GC		0.34 ± 0.07	59.39 ± 5.34	599.45 ± 215.33	2.85 ± 0.54
HQ		0.31 ± 0.03	213.09 ± 34.29	1171.87 ± 342.39	1.58 ± 0.14
GQD	**39**	0.86 ± 0.15	34.76 ± 34.18	500.16 ± 233.12	5.73 ± 2.20
GQD-GC		0.74 ± 0.29	24.09 ± 8.38	186.67 ± 129.20	2.44 ± 1.45
GG		1.12 ± 0.29	41.22 ± 11.40	139.01 ± 52.37	1.21 ± 0.24
GQD	**40**	14.00 ± 3.22	371.14 ± 66.69	8481.08 ± 1298.09	7.96 ± 2.56
GC		13.38 ± 0.78	359.33 ± 37.81	9630.73 ± 777.87	6.87 ± 2.47
GQD	**41**	0.52 ± 0.26	10.93 ± 9.40	37.47 ± 21.08	7.98 ± 2.88
GQD-GC		0.53 ± 1.20	11.07 ± 4.41	18.89 ± 4.09	6.45 ± 5.16
GG		0.52 ± 1.11	14.87 ± 9.76	67.34 ± 28.69	9.31 ± 2.19
GQD	**42**	6.42 ± 0.34	49.92 ± 6.41	1023.55 ± 117.36	8.33 ± 6.32
GQD-GC		6.34 ± 7.34	134.1 ± 3.83	1205.76 ± 259.39	4.43 ± 4.87
HQ		11.93 ± 3.34	90.81 ± 3.41	1864.9 ± 657.76	8.20 ± 1.08

*n.a. the pharmacokinetic parameters are not available due to limited amounts of data points*.

For most flavone *O*-glucuronides, flavone *C*-glycosides, and free flavones in Huang-Qin, GQD could reduce their plasma concentration. For instance, the *C*_max_ for norwogonin 7-*O*-gluA (**14**) and baicalin (**17**) in the GQD group decreased by at least 30% (GQD: **14**, 379.34 ± 98.54 ng/mL; **17**, 1183.39 ± 870.40 ng/mL. Huang-Qin: **14**, 547.17 ± 73.77 ng/mL; **17**, 4043.24 ± 584.50), and *AUC*_last_ decreased remarkably when compared with the Huang-Qin group (GQD: **14**, 5267.25 ± 987.31 h·ng/mL, **17**, 22274.24 ± 11236.50 h·ng/mL; Huang-Qin: **14**, 10951.14 ± 2530.70 h·ng/mL, **17**, 52619.53 ± 35547.30 h·ng/mL).

GQD also improved the plasma concentrations of flavonoid glycosides from Gan-Cao, including **26**, **27**, **33**, and **34**, as well as free phenolic compounds **30** and **32** (Table 1 and Figure S2). Particularly, the *AUC* of glycycoumarin (**30**) was 37.91±29.93 h·ng/mL, comparing to not detected in Gan-Cao (Figure 3). Glycycoumarin possess significant hepatoprotective and anti-liver cancer activities, but suffers from low bioavailability (Song et al., 2016). Although licorice extract could improve the bioavailability of glycycoumarin, GQD seems to be more effective (Qiao et al., 2012). On the other hand, GQD hardly changed the pharmacokinetics of glycyrrhizic acid (**36**) or its aglycone (**40**).

### Glycyrrhizic acid improved the bioavailability of berberine by inhibiting P-gp

Since Gan-Cao could remarkably improve the bioavailability of Huang-Lian alkaloids, we intend to find out the specific compounds that played this role, and to elucidate the related mechanisms. Berberine (BER, **24**) is the most abundant bioactive compound of Huang-Lian, and liquiritin (**26**) and glycyrrhizic acid (GLY, **36**) are the most abundant compounds of Gan-Cao. Thus we fed rats with BER together with GLY or liquiritin, and determined the plasma concentrations of BER. Both Gan-Cao and GLY could remarkably increase the *AUC* and *C*_*max*_ of BER, but liquiritin showed little effect (Figure S3). This indicated glycyrrhizic acid, but not liquiritin, played an important role in improving the bioavailability of berberine. It was further confirmed by conducting a pharmacokinetic study of BER with GLY. The results showed that GLY increased the *AUC*_last_ of berberine by around 6 folds (BER: *C*_max_ 12.16 ± 4.85 ng/mL, *AUC*_last_ 32.21 ± 10.78 h·ng/mL; BER+GLY: *C*_*max*_ 117.12 ± 90.22 ng/mL, *AUC*_last_ 185.30 ± 89.91 h·ng/mL) (Figure S4), and obviously slowed down the elimination (BER: *T*_1/2_ 7.53 ± 1.65 h; BER+GLY: *T*_1/2_ 8.47 ± 2.90 h).

**Figure 4:**
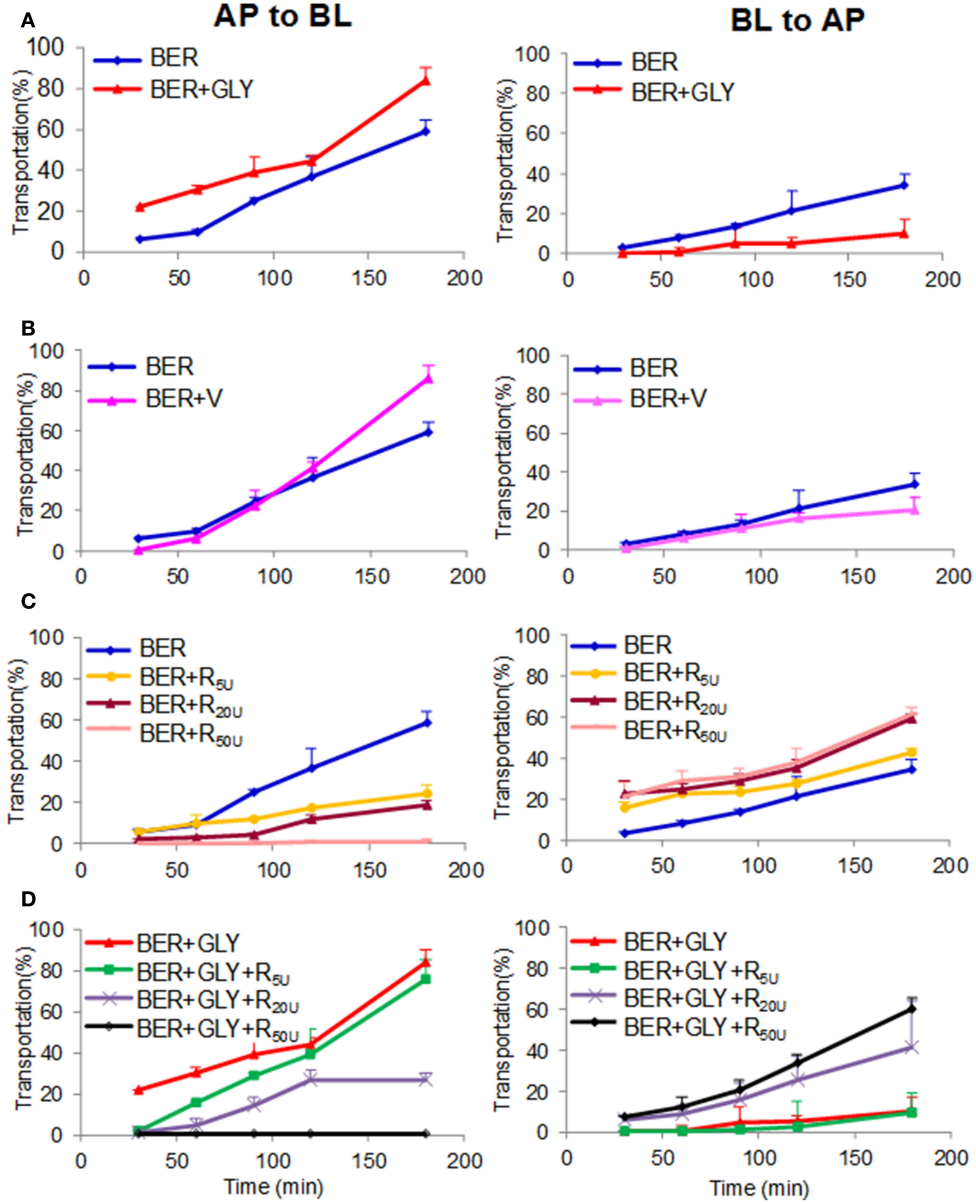
Effects of glycyrrhizic acid (GLY, 5 μM, A), verapamil (V, 5 μM, B), and rifampicin (R, 5, 20, and 50 μM, C/D) on the transportation of berberine (BER, 5 μM) through the Caco-2 cell monolayer. *P*_AB_ and *P*_BA_ represents permeability index of apical→ basolateral side and basolateral→ apical side of berberine, respectively.

Intestinal absorption is a critical factor for drug bioavailability. Our results indicated that GLY could remarkably improve intestinal absorption of BER on Caco-2 cell monolayer models. The transport rate of BER with or without GLY was 84 vs. 59% for *P*_AB_ (influx, the apical to basolateral side), and 10 vs. 34% for *P*_BA_ (efflux) (Figure 4). P-glycoprotein (P-gp) is an important transporter for drug absorption. When P-gp inducer rifampicin was added together with BER, the influx of BER decreased and the efflux increased in a dose-dependent manner. When the P-gp inhibitor verapamil was added in combination with BER, the influx of BER increased and the efflux decreased. These results indicated that BER is a P-gp substrate, as suggested by previous reports (Maeng et al., 2002). GLY showed very similar impact to verapamil, and was also a P-gp inhibitor. Moreover, GLY at 5 μM could counteract the impact of 5 or 20, but not 50 μM rifampicin, especially after 150 min treatment (Figure 4). All these evidences demonstrated that GLY improved the intestinal absorption of BER by inhibiting P-gp.

### Glycyrrhizic acid improved the anti-diabetic effects of berberine

**Figure 5:**
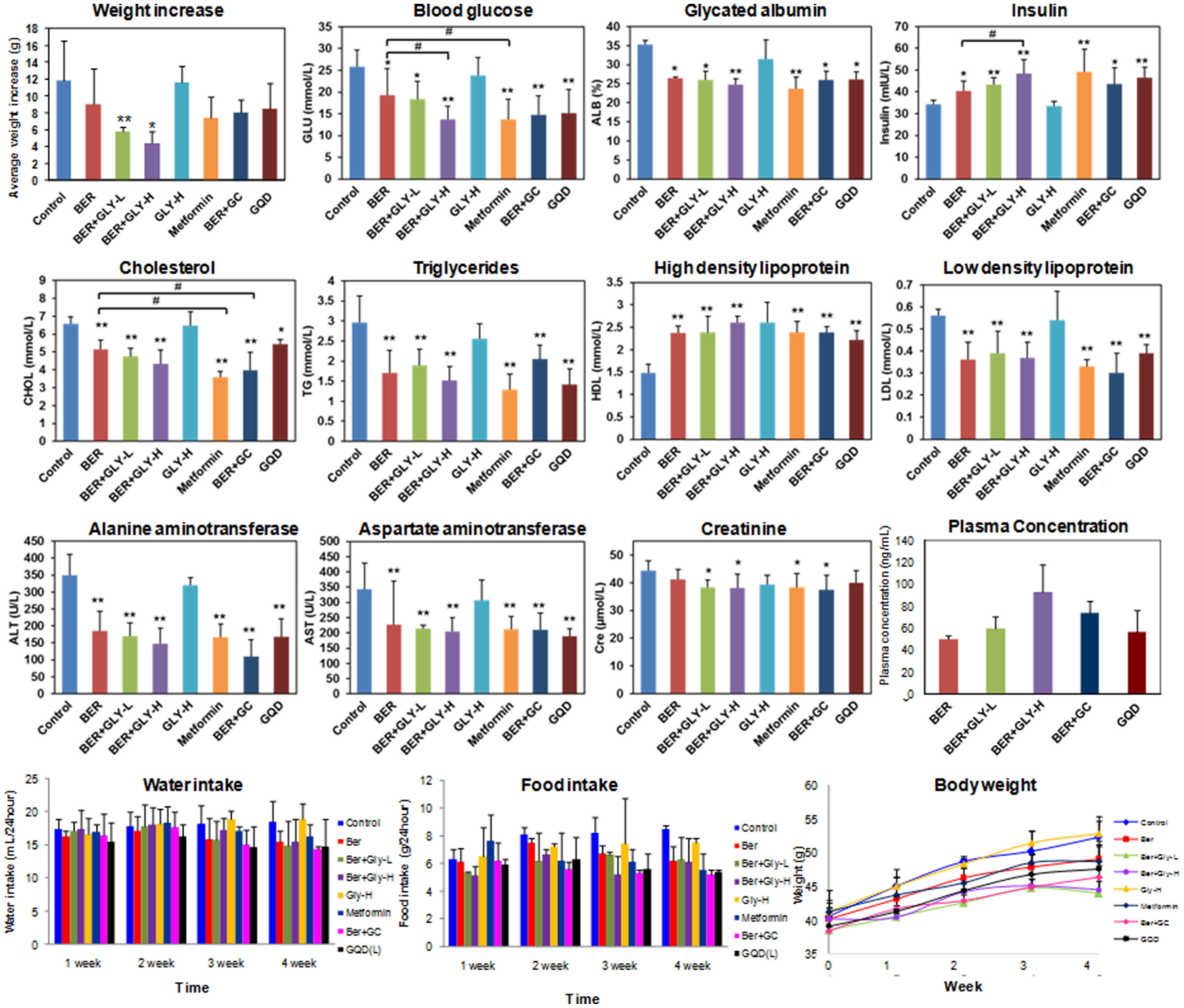
Anti-diabetic and hypolipidemic effects for the combination of berberine and glycyrrhizic acid in db/db mice. Mice serum samples were analyzed. Metformin was used as the positive control (100 mg/kg/d). Ber, berberine (50 mg/kg/d); GLY-L, glycyrrhizic acid (50 mg/kg/d); GLY-H, glycyrrhizic acid (80 mg/kg/d); GC, Gan-Cao extract (2.36 g/kg/d); GQD, Gegen-Qianlian water decoction extract (18.9 g/kg/d). *n* = 7 for the BER+GC and GQD groups; *n* = 6 for the other groups. ^*^P < 0.05; ^**^P < 0.01; ^***^P < 0.001. For the body weight panel, a significant decrease (*P* < 0.05) in 4-week body weight was observed for the positive control group and all experimental groups except for GLY-H.

BER is an anti-diabetic constituent in GQD (Lan et al., 2015). However, little is known on the role of herbal combination on its anti-diabetic effects. In this work, we fed BER (50 mg/kg/d) to spontaneous type 2 diabetic (db/db) mice for 4 weeks, and confirmed its anti-diabetic activities (Figure 5). Next we fed the mice with GLY (80 mg/kg/d), or GLY (50 mg/kg/d as low dose, 80 mg/kg/d as high dose) plus BER (50 mg/kg/d). Although GLY *per se* did not show observable anti-diabetic effects, the co-administration of GLY significantly improved the anti-diabetic activities of BER in a dose-dependent manner (Figure 5). For the high dose group, the blood glucose level decreased by 46.9%, and the serum insulin level increased by 40.8% compared to the control group.

Furthermore, the combination of GLY and BER also increased the serum concentration of high density lipoprotein, and decreased low density lipoprotein and triglycerides, indicating hypolipidemic effects. BER had been reported to possess hypolipidemic activities (Kong et al., 2004), and the combination of GLY and BER showed slightly higher activities than BER alone (Figure 5). We also noticed a liver damage in db/db mice, and BER could effectively reduce the damage by decreasing ALT and AST (Donath and Shoelson, 2011; Xie and Du, 2011). The combination of BER with GLY further decreased ALT/AST levels though the difference was not statistically significant.

Taken the above results together, positive correlations were observed between the dosage of GLY and the concentration of BER (Pearson correlation *r* = 0.75), as well as the PK-PD correlation of BER (Pearson correlation *r* = −0.66 for blood glucose, and *r* = 0.69 for insulin) (Figure S5). These results indicated GLY enhanced the anti-diabetic effects of BER, most possibly by inhibiting P-gp and thus increasing its absorption and *in vivo* exposure.

## Discussion

GQD has been used in clinical practice in China to treat type 2 diabetes. Its efficacy has been proved by clinical trials (Li et al., 2013; Xu et al., 2015). Although some of its chemical constituents like berberine had been reported to possess anti-diabetic activities, the role of GQD herbal combination on its effects remains unknown (Pang et al., 2015). Through a comprehensive multi-component PK study of GQD by monitoring 42 markers, we discovered that GLY could improve the oral bioavailability of BER by inhibiting P-gp, and thus increase the anti-diabetic effects of BER.

In this work, BER showed significant anti-diabetic and hypolipidemic activities, as consistent with previous reports (Lan et al., 2015; Liu et al., 2016). The combination of BER and GLY could further improve these activities in a dose-dependent manner. Co-administration of BER (50 mg/kg/d) and GLY (80 mg/kg/d) showed a similar efficacy to the positive control metformin (100 mg/kg/d). This potency was close to that of GQD formula, indicating GLY and BER played a key role in the anti-diabetic effects of GQD. Based on these findings, the combination of GLY and BER may be a promising natural agent for the treatment of diabetes and hyperlipidemia.

Low bioavailability has been a major hurdle for the clinical use of BER (Pang et al., 2015). This work indicated that GLY could inhibit P-gp and thus improve the bioavailability of BER. GLY had been reported to be a P-gp activator / inducer by a few research groups (Hou et al., 2012; Zheng et al., 2015). More studies indicated that GLY could inhibit P-gp (Chen et al., 2009; Bhattacharjee et al., 2015). The reason for the controversial results may be that other factors (transporters or enzymes) than P-gp were involved in the absorption and metabolism of other drugs (Feng et al., 2015). BER is mainly a P-gp substrate, and thus we obtained consistent results on cell experiments, pharmacokinetics, and pharmacodynamics.

Previous PK studies monitored no more than 12 compounds in GQD, and the effects of herbal combinations had not been revealed. In this work, 42 marker compounds were simultaneously determined. They represent majority of GQD compounds that could get into circulation after oral administration, and could be the major bioactive components of GQD (Qiao et al., 2016b). For instance, puerarin (**7**) is used to treat cerebrovascular diseases and diabetic complications (Zhou et al., 2014); flavonoids **8**, **9**, **12**, **15-17** show anti-inflammatory, anti-oxidant, and anti-viral activities (Li et al., 2011; Ji et al., 2015); alkaloids **21-25** show antihyperglycemia and antihyperlipidemia effects (Ma et al., 2016); and saponins **35** and **36** exhibit liver protective and anti-H1N1 virus activities (Li et al., 2014; Ji et al., 2016). The BER-GLY pair represents only one example of compound-compound interactions in GQD. Meanwhile, several GQD compounds were proved to be modulators or substrates of drug metabolic enzymes. They could also contribute to the pharmacokinetic alteration in GQD formula. More interactions warrant to be studied to comprehensively elucidate the multi-herb formulation mechanism of GQD.

## Conclusion

In this work, we conducted a comprehensive multi-component pharmacokinetic study of GQD by simultaneously monitoring 42 bioactive compounds in 30 min with a fully validated LC/MS/MS method. We discovered that glycyrrhizic acid could improve the oral absorption of berberine by inhibiting P-gp, and thus increase the anti-diabetic effects of berberine in db/db mice. The combination of glycyrrhizic acid and berberine may be a promising natural agent for the treatment of diabetes. This is hitherto one of the most systematic studies on the pharmacokinetics of Chinese medicine formulas.

## Ethics statement

All animal procedures were approved by Beijing Municipal Science and Technology Commission (SYXK-2011-0039, SYXK-2016-0041), and the Animal Care and Use committee of Peking University Health Science Center. All procedures were in accordance with National Institutes of Health Guide for the Care and Use of Laboratory Animals.

## Author contributions

MY, XQ, and QW participated in research design. QW, XQ, SW, YK, KL, WS, and MY conducted experiments. QW, XQ, and MY contributed new reagents or analytic tools. XQ, QW, and MY performed data analysis. MY, XQ, and QW wrote or contributed to the writing of the manuscript.

### Conflict of interest statement

The authors declare that the research was conducted in the absence of any commercial or financial relationships that could be construed as a potential conflict of interest.

## Abbreviations

BER: berberine
GC: Gan-Cao (Glycyrrhizae Radix et Rhizoma)
GG: Ge-Gen (Puerariae Lobatae Radix)
GLY: glycyrrhizic acid
GQD: Gegen-Qinlian Decoction
HL: Huang-Lian (Coptidis Rhizoma)
HQ: Huang-Qin (Scutellariae Radix)
LC/MS/MS: liquid chromatography coupled with tandem mass spectrometry
PK: pharmacokinetics
SRM: selected reaction monitoring
TCM: Traditional Chinese Medicine.
