# Supplementary material for: A 42-Markers Pharmacokinetic Study Reveals Interactions of Berberine and Glycyrrhizic Acid in the Anti-diabetic Chinese Medicine Formula Gegen-Qinlian Decoction
Source: Front Pharmacol. 2018 Jun 19;9:622. doi: 10.3389/fphar.2018.00622 (PMC6018403; doi:10.3389/fphar.2018.00622)
Supplement: Supplementary file 1 [file Presentation_1.pdf]

Supplemental Data

A 42-Markers Pharmacokinetic Study Reveals Interactions of Berberine and Glycyrrhizic Acid in the Anti-diabetic Chinese Medicine Formula Gegen-Qinlian Decoction

Xue Qiao[†,Δ], Qi Wang[†,Δ], Shuang Wang[†], Yi Kuang[†], Kai Li[†], Wei Song[†], Min Ye[*,†]

Affiliation

[†] State Key Laboratory of Natural and Biomimetic Drugs, School of Pharmaceutical Sciences, Peking University, 38 Xueyuan Road, Beijing 100191, China

[*] Corresponding author. Tel.: +86 10 82801516. Fax: +86 10 82802024. E-mail address: yemin@bjmu.edu.cn (M. Ye).

Experimental

Herbal materials and extracts.

The four component herbs, Puerariae Lobatae Radix (Ge-Gen, GG), Scutellariae Radix (Huang-Qin, HQ), Coptidis Rhizoma (Huang-Lian, HL), and Glycyrrhizae Radix et Rhizoma Praeparata cum Melle (Gan-Cao, GC) were purchased from Tianheng Pharmacy. GQD was prepared by decocting the four component herbs (Ge-Gen 25.04 g, Huang-Qin 9.43 g, Huang-Lian 9.43 g, Gan-Cao 6.31 g) in 400 mL of water for three times (1.5 h, 1.5 h, 0.5 h) to obtain the extracts, followed by a pre-extraction of Ge-Gen using 200 mL of water for 0.5 h. The decoctions were combined, filtered, and concentrated in vacuum at 50 °C. GQD-GC was prepared using the same procedure, except that Gan-Cao was removed from the formula. Final concentration of the GQD-GC extract was equivalent to 1.0 g of the crude drugs per mL. For single herb extracts, the herbs were decocted in 8-fold volume of water for three times (1.5 h, 1.5 h, 0.5 h) and then concentrated. Final concentrations of the extracts were 1.0 g/mL for Ge-Gen, and 0.5 g/mL for Huang-Qin, Huang-Lian and Gan-Cao.

Animals

For pharmacokinetic study, Male Sprague-Dawley rats (220-250 g) were obtained from the Laboratory Animal Center of Peking University Health Science Center. The rats were bred in a cage (465 × 300 × 200 mm) in a breeding room at 25 °C, 60 ± 5% humidity, and a 12-h dark-light cycle for 3 days. The rats had free access to tap water and soy-free custom diet (Ke'ao Xieli Co.). All procedures were in accordance with the National Institutes of Health *Guide for the Care and Use of Laboratory Animals* (National Research Council, 2011).

For anti-diabetic study, Eight-week-old male db/db mice (30–35 g) were purchased from Card Vince Laboratory animal Co., Ltd. The mice were bred in a cage (318 × 202 × 135 mm) in a breeding room at 25 °C, 60 ± 5% humidity, and a 12-h dark-light

cycle. The mice were given access to tap water and normal chow *ad libitum*. The animals were bred for a 2-day acclimation, and then fasted overnight before the experiments.

Experimental details for the Caco-2 model

1. Chemicals and reagents

Human colon adenocarcinoma Caco-2 cell line was purchased from the Cell Resource Center, Peking Union Medical College (Beijing, China). The cells were identified using short tandem repeat analysis. Mycoplasma contamination was examined by PCR assay. Fetal bovine serum (FBS) was purchased from PAA Laboratories GmbH (Linz, Austria). Ethylene diamine tetraacetic acid (EDTA) and dimethyl sulfoxide (DMSO) were purchased from Sigma-Aldrich (St. Louis, MO, USA). Non-essential amino acids, L-glutamine and trypsin-EDTA (0.25% (*w*/*w*) trypsin/1 mM EDTA) were from Gibco Laboratories (Life Technologies Inc., USA). Penicillin and streptomycin solutions (10,000 *U*/mL penicillin and 10,000 mg/mL streptomycin) and Hank's Balanced Salts Solution (HBSS) were obtained from M&C Gene Technology Co., Ltd. (Beijing, China). Verapamil and rifampicin were from Yuanye Biotechnology Co., Ltd. (Shanghai, China). HEPES was purchased from MP Biomedicals, LLC (Santa Ana, USA). Transwell^{TM} plates (12 wells, 12-mm membrane diameter, 3.0-µm pore size, 1.12 cm^2 surface area) and 96-well plates were from Corning Costar (Cambridge, MA, USA).

2. Caco-2 cell monolayer permeability test

Caco-2 cells were purchased at passage 18 and all experiments were performed during passages 24–35 following our previously reported method (Wang et al., 2015). Briefly, the cells were grown in DMEM with 10% FBS (inactivated at 56 °C for 30 min), 1% NEAA, and 1% penicillin and streptomycin solution in a 37 °C incubator with 5% CO_2. For the bidirectional transport experiments, the GQD, GQD_{-GC}, and Huang-Lian water extracts were respectively freeze-dried to obtain powder, dissolved in DMSO, and then diluted with HBSS to prepare the extract solutions (32, 28, and 6 µg/mL, respectively). Berberine was dissolved in DMSO, and then diluted with HBSS

containing glucose (25 mM) and HEPES (10 mM). The final concentration of berberine was 5 μM. The final concentration of DMSO was below 1% (*v*/*v*).

Verapamil (5 μM) and rifampicin (5, 10, and 20 μM) were used as P-gp inhibitor and inducer of Caco-2 cells, respectively. For the inhibitor experiments, the Caco-2 cells were cultivated in DMEM until the 21st day. After removal of the medium, the cells were incubated for 30 minutes with HBSS containing 5 μM of verapamil. For the inducer experiments, the cells were cultivated in DMEM until the 18th day, and then cultivated in DMEM containing rifampicin (5, 10, and 20 μM for 24 h). The medium was removed on the 21st day, and the cells were then incubated for 30 min with HBSS. Berberine and glycyrrhizic acid were added to the system following HBSS incubation.

For all bidirectional transport assays, samples (300 μL) were collected after 30, 60, 90, 120, 150 and 180 min of incubation, and the culture was immediately replenished with an equal volume of HBSS. The samples were freeze-dried and then dissolved in 300 μL of methanol. The solutions were filtered through 0.22-μm membranes before LC/MS/MS analysis.

3. Inhibitor and inducer experiments

Verapamil and rifampicin were used as P-gp inhibitor and inducer of Caco-2 cells, respectively. For the inhibition experiments, Caco-2 cells were cultivated in DMEM until the 21st day. After removal of the medium, the cells were incubated for 30 minutes with HBSS containing verapamil (5 μM). For induction experiments, the cells were cultivated in DMEM until the 18th day, and then cultivated in DMEM containing rifampicin (5, 10, and 20 μM) for 24 h. The medium was removed on the 21st day, and the cells were then incubated for 30 min with HBSS. Berberine and glycyrrhizic acid were added to the system following HBSS incubation.

4. Sample preparation

For all bidirectional transport assays, samples (300 μL) were collected after 30, 60, 90, 120, 150 and 180 min of incubation, and the culture was immediately replenished with an equal volume of HBSS. The samples were freeze-dried and then dissolved in 300 μL of methanol. The solutions were filtered through 0.22-μm membranes before LC/MS/MS analysis.

Experimental details for the PK study

1. Preparation of internal standard solution, calibration standard solutions, and quality control solutions

Butein 4-*O*-glucoside (0.1 µg/mL) was dissolved in 67% methanol to prepare the internal standard solution. Reference standard compounds were dissolved in the internal standard solution to prepare individual stock solutions (1.0 mg/mL). These stock solutions were mixed to form a standard solution, containing 23800 ng/mL of each compound. The standard solution was then serially diluted to obtain calibration standard solutions (11900, 5950, 2975, 1190, 595, 297.5, 119, 59.5, 29.75, 11.9, 5.95, 2.975, and 1.19 ng/mL for each compound). Quality control stock solutions were prepared at three concentration levels as high QC (HQC), middle QC (MQC), and low QC (LQC), based on linear ranges of the analytes.

2. Sample preparation

2.1. Sample preparation for pharmacokinetic study

The calibration standard and quality control solutions for pharmacokinetic study were prepared with the same procedures for plasma samples as described below. For these samples, 150 µL of stock solution was evaporated to dryness under a gentle stream of nitrogen, and then 150 µL of blank rat plasma, 150 µL of internal standard solution, and 300 µL of acetonitrile were added. The mixture was vortexed (2200 rpm) for 2 min, and ultrasonicated in a water bath for 5 min. Then, the mixture was vortexed for another 2 min, and centrifuged (9000 rpm, 4 °C) for 10 min. The supernatant was separated and evaporated to dryness at 37 °C using a speedvac concentrator. The residue was stored at -80 °C and redissolved in 150 µL of 67% methanol before analysis. All samples were filtered through 0.22-µm membranes. A 5-µL aliquot was injected for LC/MS/MS analysis.

2.2. Sample preparation for anti-diabetic activity study

On day 28, db/db mice were fasted for 12 h, and blood was collected and centrifuged to obtain serum for insulin and biochemical tests. After that, 150 μL of plasma was prepared by adding 150 μL of internal standard solution, 150 μL of methanol, and 300 μL of acetonitrile. The processing method was consistent with sample preparation for pharmacokinetic study.

3. Method optimization

3.1. Optimization of chromatographic separation

Different solvents (67% methanol, and 100% methanol) were compared to effectively extract both hydrophilic and hydrophobic compounds of GQD. 67% methanol showed better extraction recovery for most compounds. A number of columns were tested, and Waters XTerra C_{18} column was selected to obtain the best peak shapes and chromatographic separation of the 42 markers. Different organic HPLC mobile phases (acetonitrile, and acetonitrile containing 2% (*v*/*v*) methanol) were compared. Good peak shapes were obtained when acetonitrile containing 2% (*v*/*v*) methanol was used.

3.2. Optimization of MS/MS conditions

(+)-ESI mode was used to obtain high ionization efficiency of alkaloids. To optimize MS parameters, 42 pure compounds were individually injected into (+)-ESI source by continuous infusion. MS fragment ions, tube lens offset voltage, and collision energy (CE) were optimized for all the 42 analytes. The optimized parameters are given in Table S1. To ensure the precision of sample extraction and MS detection, butein 4-*O*-glucoside was employed as internal standard.

TABLE S1. SRM transitions of 42 GQD compounds for PK analysis.

No.	Analyte	RT	Parent ion	quantitative			qualitative		
				Product ion	CE	Tube Lens	Product ion	CE	Tube Lens
		min	*m/z*	*m/z*	V	V	*m/z*	V	V
**1**	Daidzin	10.14	417	255	40	115	137	40	120
**2**	3'-Methoxypuerarin	9.4	447	327	30	130	297	35	120
**3**	Daidzein	16.74	255	227	32	115	197	40	85
**4**	Genistin	11.85	433	271	30	130	153	45	152
**5**	Formononetin 8-*C*-apiofuranosyl (1,6)glucoside	12.12	563	311	30	130	149	45	120
**6**	Genistein 8-*C*-apiofuranosyl(1,6)glucoside	10.44	565	313	30	115	433	20	95
**7**	Puerarin	9.07	417	297	35	130	257	35	135
**8**	Wogonin	21.97	285	270	32	115	151	45	98
**9**	Oroxylin A	22.46	285	270	32	115	168	40	85
**10**	Chrysin 6-*C*-arabinoside-8-*C*-glucoside	11.74	549	429	40	130	393	25	115
**11**	Chrysin-8-*C*-arabinoside-6-*C*-glucoside	11.16	549	387	40	115	363	30	95
**12**	Baicalein	20.65	271	227	42	115	123	50	120
**13**	Wogonin 5-*O*-glucoside	17.1	447	285	40	115	270	35	80
**14**	Norwogonin 7-*O*-glucuronide	16.01	447	271	42	152	286	30	113
**15**	Oroxylin A 7-*O*-glucuronide	17.79	461	285	40	115	270	45	120
**16**	Wogonoside	18.32	461	285	40	115	270	50	85
**17**	Baicalin	17.14	447	271	42	152	123	50	180
**18**	Chrysin	22.03	255	153	40	115	103	50	145
**19**	Magnoflorine	9	342	297	43	45	265	30	40
**20**	Demethyleneberberine	11.18	324	309	40	106	280	30	80
**21**	Coptisine	12.64	320	292	40	45	204	45	115

**22**	Epiberberine	12.57	336	320	42	106	292.1	45	115
**23**	Jatrorrhizine	12.85	338	323	40	45	294	30	35
**24**	Berberine	15.67	336	320	42	106	292	30	80
**25**	Palmatine	15.39	352	293	40	106	336	20	80
**26**	Liquiritin	11.41	419	257	35	106	114	25	40
**27**	Isoliquiritin	15.46	419	257	35	106	137	50	115
**28**	Isoliquiritigenin	20.2	257	137	40	130	147	30	110
**29**	Liquiritigenin	11.45	257	137	40	130	147	35	115
**30**	Glycycoumarin	22.28	369	313	35	145	285	25	115
**31**	Glycyrol	23.56	367	309	35	145	281	20	115
**32**	Formononetin	20.68	269	254	32	115	197	40	120
**33**	Liquiritin apioside	11.21	551	257	32	99	257.1	20	80
**34**	Isoliquiritin apioside	14.27	551	257	32	99	137	45	117
**35**	Licorice-saponin G2	20	839	469	37	240	353	40	240
**36**	Glycyrrhizic acid	20.57	823	453	34	214	471	30	165
**37**	3'-Methoxymirificin	9.42	579	327	30	130	297	40	130
**38**	Lateriflorein 7-*O*-glucuronide	14.04	477	301	40	115	286	50	87
**39**	Ononin	15.96	431	269	20	45	254	35	50
**40**	Glycyrrhetinic acid	27.22	471	189	43	211	317	30	99
**41**	(4S)-Puerol B 2″-*O*-glucopyranoside	14.52	475	107	33	180	313	25	40
**42**	Chrysin 7-*O*-glucuronide	17.84	431	255	42	152	269	30	115
IS	Butein-4-*O*-glucoside	14.6	435	273	24	152	137	50	135

Programmed SRM segments: A, 6-11.12 min (analytes **1**, **2**, **6**, **7**, **11**, **19**, **20**, **33**, **37**); B, 11.12-15.27 min (analytes **4**, **5**, **6**, **10**, **11**, **20**, **21**, **22**, **23**, **26**, **29**, **33**, **34**, **38**, **41**, IS); C, 15.27-18.05 min (**3**, **13**, **14**, **15**, **16**, **17**, **24**, **25**, **27**, **34**, **38**, **39**, **41**, **42**, IS); D, 18.05-21.13 min (**12**, **15**, **16**, **28**, **32**, **35**, **36**, **42**); E, 21.13-31 min (**8**, **9**, **12**, **18**, **30**, **31**, **32**, **36**, **40**).

4. Method validation

The method was validated in terms of specificity, linearity, accuracy, precision, matrix effect, extraction efficiency, and stability in accordance with the USA Food and Drug Administration (FDA) bioanalytical method validation guidance (U.S. Food and Drug Administration, 2013).

4.1. Specificity

The specificity of the method was investigated by analyzing blank plasma samples from six rats. The chromatogram of each blank plasma sample was tested for interference using the proposed extraction procedure and compared with the spiked rat plasma. As a result, no significant endogenous interference was observed in the blank rat plasma (Fig. S1).

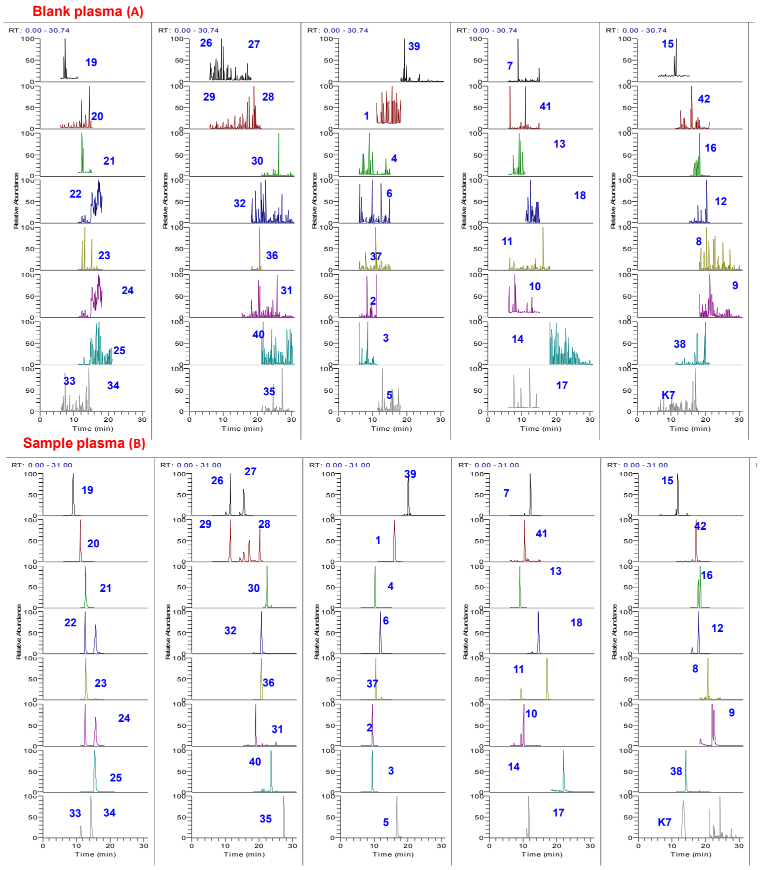

Fig. S1. SRM chromatograms of 42 analytes and the internal standard in blank plasma (A) and sample plasma collected at 1 h (B). K7 refers to the internal standard.

4.2. Linearity

The linearity of each calibration curve was determined by plotting the analyte / internal standard peak area ratio (y) against the concentrations of analytes (x). All the calibration curves showed good linearity with correlation coefficients (r^2) of >0.9900 (Table S2). The lower limit of quantification (LLOQ) was determined at signal-to-noise ratios of 10. The LLOQs varied from 1.19-29.75 ng/mL for the analytes. The regression equations, correlation coefficient, dynamic ranges and LLOQs were listed in Table S2.

TABLE S2. Linear regressions of the 42 analytes.

Analyte	Regression equation (y=ax+b)	Weighting power	Correlation coefficient	Linear range (ng/mL)	LLOQ (ng/mL)
**1**	$y = 0.014+0.011\,x -4.61\times10^{-7}x^2$	1/x	0.9923	1.19-11900	1.19
**2**	$y = 0.021+0.0050\,x$	1/x	0.9907	2.975-11900	2.975
**3**	$y = 0.025+0.013\,x -4.52\times10^{-7}x^2$	1/x	0.9940	1.19-11900	1.19
**4**	$y = 0.016+0.012\,x$	1/x	0.9909	2.975-11900	2.975
**5**	$y = 0.0086+0.010\,x$	1/x	0.9942	1.19-11900	1.19
**6**	$y = -8.6\times10^{-4}+0.0045\,x$	1/x	0.9934	2.975-5950	2.975
**7**	$y = 0.0073+0.0036\,x$	1/x	0.9913	1.19-11900	1.19
**8**	$y = 0.84+0.16\,x$	1/x	0.9909	1.19-11900	1.19
**9**	$y = 0.96+0.16\,x$	1/x	0.9914	1.19-11900	1.19
**10**	$y = 3.01\times10^{-4}+1.89\times10^{-4}x$	1/x	0.9908	5.95-11900	5.95
**11**	$y = 9.11\times10^{-4}+6.32\times10^{-4}x$	1/x	0.9900	5.95-11900	5.95
**12**	$y = 0.0036+7.77\times10^{-4}x$	1/x	0.9906	1.19-11900	1.19
**13**	$y = -2.29\times10^{-4}+0.0038\,x$	1/x	0.9914	1.19-11900	1.19
**14**	$y = 0.021+0.015\,x -6.691\times10^{-7}x^2$	1/x	0.9909	2.975-11900	2.975
**15**	$y = 0.0017+0.0083\,x$	1/x	0.9925	1.19-11900	1.19
**16**	$y = 0.0182+0.012\,x$	1/x	0.9912	1.19-11900	1.19
**17**	$y = 0.0181+0.015\,x -7.70\times10^{-7}x^2$	1/x	0.9913	1.19-11900	1.19
**18**	$y = 0.044+0.018\,x$	1/x	0.9924	1.19-11900	1.19
**19**	$y = -0.0025+3.70\times10^{-4}x -1.68\times10^{-8}x^2$	1/x	0.9912	29.75-11900	29.75
**20**	$y = 0.75208+0.037\,x$	1/x	0.9912	11.90-5950	11.90
**21**	$y = 0.018+0.012x -5.36\times10^{-7}x^2$	1/x	0.9909	1.19-11900	1.19
**22**	$y = 0.57+0.26\,x -1.23\times10^{-5}x^2$	1/x	0.9929	1.19-11900	1.19
**23**	$y = -0.053+0.041\,x -1.33\times10^{-6}x^2$	1/x	0.9903	1.19-11900	1.19
**24**	$y = 1.15+0.23\,x -8.39\times10^{-7}x^2$	1/x	0.9908	1.19-11900	1.19
**25**	$y = -0.0035+0.0021\,x$	1/x	0.9978	2.975-11900	2.975
**26**	$y = 0.0012+0.00145\,x$	1/x	0.9924	2.975-11900	2.975

**27**	$y = 4.51\times10^{-4}+0.0022x$	1/x	0.9960	2.975-11900	2.975
**28**	$y = 0.022+0.018x$	1/x	0.9946	1.19-11900	1.19
**29**	$y = 0.023+0.0077x$	1/x	0.9922	1.19-11900	1.19
**30**	$y = 0.0052+0.0026x -1.08\times10^{-7}x^2$	1/x	0.9913	1.19-11900	1.19
**31**	$y = -1.82\times10^{-4}+0.0015x$	1/x	0.9920	2.975-11900	2.975
**32**	$y = 0.091+0.062x -1.69\times10^{-6}x^2$	1/x	0.9908	1.19-11900	1.19
**33**	$y = 1.88\times10^{-4}+0.0034x$	1/x	0.9934	1.19-11900	1.19
**34**	$y = 0.0031+0.011x$	1/x	0.9942	2.975-11900	2.975
**35**	$y = 0.084+0.0040x$	1/x	0.9918	11.90-11900	11.90
**36**	$y = 0.14+0.025x -1.08\times10^{-6}x^2$	1/x	0.9958	5.95-11900	5.95
**37**	$y = -0.0039+0.0065x -1.29\times10^{-7}x^2$	1/x	0.9903	2.975-11900	2.975
**38**	$y = 0.0058+0.0073x$	1/x	0.9956	1.19-11900	1.19
**39**	$y = 0.0040+0.0060x -1.16\times10^{-7}x^2$	1/x	0.9948	2.975-11900	2.975
**40**	$y = -0.0034+0.0020x$	1/x	0.9978	2.975-11900	2.975
**41**	$y = 0.0023+0.0047x$	1/x	0.9945	2.975-11900	2.975
**42**	$y = 0.0060+0.017x -5.67\times10^{-7}x^2$	1/x	0.9931	1.19-11900	1.19

4.3. Intra- and inter-day precision and accuracy

The intra- and inter-day precisions and accuracy were investigated by determining QC samples at three different concentrations (six replicates for each concentration level) in the same day for five times and on three consecutive days. The precision of the method at each QC concentration was expressed as the relative standard deviation (RSD) and the accuracy was described as relative error (RE). The RSD values of intra- and inter-day precisions ranged from 0.50% to 15.53% and 0.57% to 21.98%, respectively. The RSD and RE values of accuracy for LQC, MQC, and HQC ranged from 0.37% to 15.12% and -18.02% to 18.12%, respectively (Table S3).

TABLE S3. Intra- and inter-day variations of the 42 analytes (n = 5).

Analyte	Calc. Con. (ng/ml)	Intra-day precision		Inter-day precision		Accuracy	
		RSD (%)	Accuracy (%)	RSD (%)	Accuracy (%)	RE (%)	RSD (%)
	600.00	10.50	86.33	7.46	105.68	-7.59	8.24
**1**	300.00	8.28	84.70	10.00	95.73	5.85	6.10
	3.00	6.87	87.47	13.57	102.28	-14.15	6.44
	1000.00	1.82	116.91	5.95	114.32	16.38	1.44
**2**	500.00	4.62	106.02	3.70	113.64	16.22	3.52
	5.00	7.17	86.69	10.87	89.80	3.43	5.79
**3**	600.00	2.35	84.14	2.57	84.82	-18.02	0.37

	300.00	1.62	80.84	14.47	90.66	-11.58	3.84
	3.00	5.43	95.18	5.81	87.12	-10.20	8.28
	1000.00	1.72	119.39	7.23	111.64	16.80	0.90
**4**	500.00	2.93	111.49	3.42	116.07	17.67	0.37
	5.00	13.75	98.64	1.93	116.76	8.05	11.78
	600.00	3.74	110.62	1.98	116.89	12.78	2.47
**5**	300.00	1.80	117.32	1.62	116.34	15.42	1.29
	3.00	12.07	80.43	7.22	97.53	-11.14	5.89
	500.00	13.60	114.47	4.48	113.94	11.80	3.91
**6**	250.00	10.00	109.61	10.56	99.65	-5.55	12.86
	5.00	11.91	114.21	17.97	104.03	-6.53	13.94
	600.00	6.26	119.53	2.63	116.44	9.24	3.61
**7**	300.00	1.47	116.69	4.64	113.18	10.75	5.56
	3.00	4.01	108.08	15.87	105.29	4.67	12.38
	600.00	8.17	107.70	6.13	111.79	-5.87	9.37
**8**	300.00	6.01	112.54	13.64	105.70	-4.27	8.12
	3.00	4.60	89.44	5.22	86.61	-6.41	4.53
	600.00	4.84	109.83	12.86	94.11	-10.43	6.39
**9**	300.00	7.35	91.33	11.06	98.37	-11.79	4.86
	3.00	6.11	88.28	13.13	92.64	-3.16	6.83
	1000.00	5.29	111.21	11.35	101.39	7.30	12.10
**10**	500.00	8.36	109.06	15.30	105.16	6.91	11.97
	10.00	4.75	93.34	10.97	106.19	-1.72	14.83
	1000.00	3.66	109.93	2.06	114.09	16.17	1.38
**11**	500.00	4.12	109.50	2.07	117.61	11.59	4.50
	10.00	10.10	89.78	5.04	111.28	-5.56	11.19
	600.00	12.43	82.75	7.05	99.72	-2.53	5.81
**12**	300.00	9.91	112.17	13.28	97.74	4.98	10.35
	3.00	7.79	109.68	7.80	101.93	-4.59	9.86
	600.00	11.49	92.21	11.99	104.35	16.72	0.54
**13**	300.00	8.43	99.62	8.45	106.81	17.17	0.97
	3.00	11.23	89.68	5.42	100.89	-2.25	8.93
	1000.00	9.80	104.05	14.38	108.25	16.26	1.34
**14**	500.00	3.82	108.93	9.00	107.83	18.12	1.22
	5.00	10.41	104.15	21.98	93.41	-4.60	11.89
	600.00	4.24	116.27	17.56	107.69	14.71	3.70
**15**	300.00	1.13	119.98	5.80	114.94	16.79	1.62
	3.00	6.40	119.47	4.09	113.56	6.29	8.06
	600.00	1.86	116.77	12.20	109.04	14.85	1.43
**16**	300.00	2.99	116.02	1.37	116.58	17.29	0.98
	3.00	4.10	115.38	2.25	107.50	9.37	4.52
	600.00	10.11	86.83	18.38	102.96	15.75	1.25
**17**	300.00	8.43	93.83	11.98	102.85	16.72	1.02
	3.00	11.75	119.74	4.41	98.98	1.61	4.36

	600.00	14.17	115.27	6.81	110.70	12.38	6.21
**18**	300.00	1.68	119.77	1.12	118.76	12.66	5.94
	3.00	6.52	102.36	3.18	106.34	-0.46	4.47
	1000.00	13.92	80.51	3.66	88.90	-3.61	6.06
**19**	500.00	8.87	92.72	5.83	105.77	1.94	10.76
	50.00	6.82	80.01	12.78	109.64	-3.58	13.94
	500.00	10.75	94.41	12.10	103.85	-4.20	12.45
**20**	250.00	6.69	90.04	18.74	97.18	-12.96	4.65
	25.00	11.66	81.54	13.57	97.05	8.28	10.44
	600.00	5.58	94.17	6.90	98.82	-4.90	0.87
**21**	300.00	7.91	82.19	11.19	87.00	-14.36	4.69
	3.00	15.53	84.25	15.12	92.58	7.52	14.05
	600.00	2.26	85.41	15.74	94.53	-9.94	6.80
**22**	300.00	12.66	108.95	1.66	109.80	0.42	11.59
	3.00	8.67	91.23	6.70	107.97	4.58	9.82
	1000.00	6.65	91.88	11.21	94.49	-17.22	1.26
**23**	500.00	6.45	86.87	15.79	97.13	-3.99	6.90
	5.00	5.66	107.85	4.74	113.32	10.87	6.73
	600.00	4.57	119.93	11.18	109.71	-8.67	2.89
**24**	300.00	1.74	111.09	10.73	108.60	3.20	6.28
	3.00	4.45	105.84	4.18	107.88	8.21	2.27
	1000.00	2.45	110.66	6.15	108.97	-4.62	4.83
**25**	500.00	3.17	111.01	9.39	108.93	5.28	5.94
	5.00	3.44	87.79	0.91	88.10	-7.51	7.76
	1000.00	6.66	94.27	5.50	93.38	-4.17	6.94
**26**	500.00	5.88	91.52	4.69	96.71	-3.55	1.90
	5.00	5.35	98.85	5.09	103.54	-0.50	6.99
	1000.00	8.16	84.63	16.19	102.78	-3.10	7.50
**27**	500.00	4.82	87.22	6.09	95.42	7.91	6.16
	5.00	4.81	90.56	7.52	88.76	-11.55	5.66
	600.00	5.37	98.49	12.63	102.83	-8.14	2.37
**28**	300.00	3.80	97.40	3.86	96.53	-1.91	4.28
	3.00	3.64	82.56	2.68	89.50	-12.17	6.35
	600.00	0.50	119.50	14.91	107.30	5.91	1.24
**29**	300.00	2.35	111.74	1.67	110.44	12.71	2.39
	3.00	9.49	105.70	0.57	93.16	-4.89	12.51
	600.00	8.59	107.40	20.70	106.02	-0.77	2.64
**30**	300.00	9.28	111.76	13.10	105.62	-6.50	9.85
	3.00	11.10	83.70	19.04	98.15	0.85	8.25
	1000.00	2.44	111.94	1.12	116.92	9.42	7.19
**31**	500.00	1.74	116.84	2.01	111.34	9.46	5.08
	5.00	4.46	104.82	13.45	102.50	-5.48	7.06
**32**	600.00	5.68	95.57	2.54	87.56	-8.71	4.43
	300.00	6.24	92.09	10.49	91.27	-4.72	7.19

	3.00	5.83	91.21	7.10	87.59	-12.93	9.21
	600.00	2.44	116.35	5.64	112.97	11.79	4.28
**33**	300.00	2.79	119.17	0.88	118.08	17.05	1.50
	3.00	1.37	114.84	11.97	110.75	15.18	2.62
	1000.00	10.54	92.18	1.91	103.74	-2.59	7.14
**34**	500.00	8.42	89.33	5.14	94.92	6.70	8.21
	5.00	7.01	92.64	17.19	95.57	5.53	9.24
	1000.00	1.56	117.43	1.87	115.29	16.77	2.10
**35**	500.00	1.01	118.61	3.13	115.38	16.30	0.76
	25.00	5.62	109.13	12.95	96.71	0.19	12.77
	600.00	6.93	106.43	2.85	116.60	11.94	2.96
**36**	300.00	3.78	112.03	1.51	113.78	16.34	2.24
	15.00	4.45	106.85	2.76	113.56	0.34	11.53
	1000.00	4.44	110.61	1.91	104.60	7.87	5.64
**37**	500.00	5.51	103.08	7.34	111.70	8.86	9.91
	5.00	5.29	83.47	12.14	106.12	-4.90	15.12
	600.00	4.00	112.46	15.63	101.31	5.85	6.94
**38**	300.00	8.95	100.00	8.83	109.26	12.32	3.31
	3.00	7.45	108.90	9.95	93.99	-0.55	10.74
	1000.00	8.01	90.18	7.61	106.80	6.33	7.96
**39**	250.00	6.06	102.77	6.75	105.20	3.80	4.23
	5.00	5.75	104.23	4.00	108.26	9.02	6.83
	1000.00	11.47	93.83	5.14	111.77	11.87	3.54
**40**	500.00	10.46	91.12	6.68	97.45	8.57	6.20
	5.00	10.44	115.38	12.61	103.50	-2.02	6.50
	1000.00	6.70	95.33	19.22	103.96	-2.37	10.47
**41**	500.00	9.84	107.35	14.98	92.17	7.80	11.92
	5.00	6.55	81.54	8.30	93.95	-6.23	12.80
	600.00	2.97	112.60	0.87	115.97	16.06	1.47
**42**	300.00	0.91	117.77	1.82	117.02	17.95	0.87
	3.00	1.53	118.03	1.85	115.86	15.44	2.16

Note: RE (%) = [(the mean value of the observed concentration – nominal concentration) / (nominal concentration)] × 100. RSD, relative standard deviation in %.

4.4. Recovery and matrix effect

The extraction recoveries and the matrix effects of analytes were determined as we had previously reported (Qiao *et al.*, 2012). The method recoveries were determined by comparing the nominal concentration of plasma QC samples to the measured concentration of QC samples (analytes were diluted in methanol). The extraction and method recoveries at three different QC concentrations were between 82.60%–118.68% and 81.56%–118.67%, suggesting that the method was accurate (Table S4). The

matrix effects were not significant for most analytes, with ion suppression ranged from -11.34% to 7.33% (Table S4).

TABLE S4. Recovery and matrix effect of the analytes (n = 3).

Analyte	Calc. Con. (ng/ml)	Extraction Recovery		Method Recovery		Matrix effects	
		Average (%)	RSD (%)	Average (%)	RSD (%)	Average (%)	RSD (%)
**1**	600.00	92.33	8.15	91.60	8.26	101.75	5.54
	300.00	102.57	2.69	109.68	5.91	100.23	12.70
	3.00	101.70	0.92	85.85	6.44	100.08	2.22
**2**	1000.00	103.70	4.10	117.51	1.58	100.19	3.64
	500.00	103.76	2.64	116.22	3.52	100.21	5.49
	5.00	105.89	4.18	107.59	5.23	100.68	7.36
**3**	600.00	88.45	8.85	81.56	0.95	101.74	1.88
	300.00	94.28	5.98	88.00	4.20	101.24	10.29
	3.00	96.40	1.98	86.94	8.71	103.01	6.17
**4**	1000.00	99.89	1.25	117.63	0.94	100.43	0.39
	500.00	101.17	3.89	117.98	0.52	99.54	1.24
	5.00	113.67	8.56	106.77	10.84	100.02	5.37
**5**	600.00	97.96	1.61	114.76	2.55	100.26	1.24
	300.00	100.85	1.15	116.18	1.37	100.45	0.37
	3.00	92.74	9.31	88.29	6.65	98.17	0.92
**6**	500.00	109.49	8.19	106.46	14.04	106.47	7.11
	250.00	103.96	6.83	94.45	12.86	101.01	6.82
	5.00	92.84	4.47	91.81	15.51	100.24	1.17
**7**	600.00	98.09	4.89	109.24	3.61	102.23	3.11
	300.00	101.69	1.40	110.67	5.44	100.18	1.47
	3.00	106.56	3.99	106.20	12.88	111.03	1.75
**8**	600.00	86.29	4.28	94.13	9.37	100.19	0.75
	300.00	82.60	6.72	95.73	8.12	101.39	3.60
	3.00	97.38	6.72	93.30	4.81	100.23	2.04
**9**	600.00	83.73	3.10	88.61	7.13	101.09	1.11
	300.00	83.80	1.52	85.68	5.32	99.98	6.37
	3.00	95.72	2.99	96.84	6.83	97.09	2.46
**10**	1000.00	100.71	4.80	113.38	7.60	100.88	2.68
	500.00	101.72	6.49	107.39	12.18	99.39	0.62
	10.00	98.57	3.99	95.11	10.89	102.63	7.90
**11**	1000.00	101.66	1.26	117.06	1.55	101.09	0.80
	500.00	101.57	4.91	111.61	4.49	101.14	1.64
	10.00	106.90	3.02	91.84	9.32	100.27	1.76
**12**	600.00	87.20	4.18	95.10	9.36	100.04	2.02
	300.00	96.57	5.37	110.54	6.26	103.29	7.16

	3.00	99.32	3.37	91.64	12.15	98.23	2.04
	600.00	113.23	11.70	117.03	0.62	102.25	5.88
**13**	300.00	109.07	10.14	117.38	1.19	103.06	8.20
	3.00	94.63	4.97	100.58	9.18	101.26	1.43
	1000.00	107.04	9.69	116.99	1.80	103.08	3.91
**14**	500.00	111.68	8.40	118.67	1.18	100.00	11.16
	5.00	100.51	6.57	90.43	11.83	100.18	3.08
	600.00	102.06	2.95	116.89	2.25	101.59	0.94
**15**	300.00	110.91	7.53	117.44	1.84	102.22	10.98
	3.00	100.27	4.41	110.57	7.43	100.36	6.49
	600.00	98.94	0.49	115.88	1.35	101.27	0.45
**16**	300.00	105.29	4.35	118.09	1.04	99.87	10.11
	3.00	98.19	3.62	111.88	5.21	101.04	4.87
	600.00	118.68	9.75	116.17	1.48	102.37	5.64
**17**	300.00	114.61	11.94	117.64	0.88	104.69	6.21
	3.00	99.52	5.00	100.30	6.60	98.77	6.88
	600.00	105.57	8.01	106.61	15.26	100.11	1.95
**18**	300.00	99.56	1.03	115.84	2.05	100.38	1.46
	3.00	103.16	3.50	96.76	5.61	106.37	2.69
	1000.00	102.04	10.32	98.65	9.02	100.15	9.53
**19**	500.00	96.27	3.98	104.11	10.80	100.06	10.52
	50.00	97.01	3.16	96.42	13.94	101.24	16.75
	500.00	95.33	5.49	101.28	9.87	100.34	8.44
**20**	250.00	89.87	6.21	84.81	4.59	100.06	8.32
	25.00	102.47	5.90	104.17	16.16	100.17	5.39
	600.00	97.76	10.69	95.10	0.87	101.17	0.59
**21**	300.00	86.55	14.07	96.33	19.14	101.16	3.83
	3.00	106.32	3.98	114.63	4.04	100.28	2.29
	600.00	99.97	2.96	89.47	6.38	100.79	2.74
**22**	300.00	100.73	4.65	100.42	11.59	100.24	12.41
	3.00	91.29	7.67	104.58	9.82	101.93	1.29
	1000.00	89.07	8.53	82.57	1.42	100.42	3.24
**23**	500.00	94.63	2.80	96.98	5.97	100.08	11.17
	5.00	104.79	6.76	107.39	13.18	101.36	4.44
	600.00	87.88	6.85	90.31	3.90	102.28	3.51
**24**	300.00	90.38	4.74	106.63	5.80	100.16	3.68
	3.00	95.93	0.62	108.99	2.95	98.43	5.22
	1000.00	87.14	8.05	94.39	5.64	101.39	2.31
**25**	500.00	97.85	2.80	106.02	6.93	100.16	4.21
	5.00	85.12	2.39	92.03	8.67	100.99	5.43
	1000.00	102.06	3.94	95.83	6.94	100.33	1.23
**26**	500.00	102.77	1.99	96.45	1.90	101.09	4.00
	5.00	103.26	4.35	99.50	6.99	103.07	2.70
**27**	1000.00	96.18	2.73	96.90	7.50	99.78	1.45

	500.00	97.87	3.32	107.50	6.68	101.35	1.31
	5.00	101.09	2.36	88.45	5.66	102.01	1.70
	600.00	90.51	8.37	90.72	2.56	101.04	5.30
**28**	300.00	96.38	5.32	93.36	8.06	100.17	12.57
	3.00	96.30	2.01	87.63	6.65	100.38	3.81
	600.00	96.16	5.28	108.50	4.85	100.19	1.64
**29**	300.00	112.89	11.45	114.18	3.13	96.08	2.30
	3.00	97.77	1.42	87.95	5.31	98.77	4.82
	600.00	88.09	2.31	101.32	3.92	101.13	2.42
**30**	300.00	86.66	4.20	93.36	9.97	100.39	4.49
	3.00	93.34	2.86	98.41	11.24	101.34	2.68
	1000.00	96.02	2.64	112.57	3.73	99.88	1.35
**31**	500.00	95.54	0.56	112.21	1.31	101.12	2.94
	5.00	89.86	7.05	94.52	7.06	102.41	6.52
	600.00	95.14	3.43	91.29	4.43	100.00	1.44
**32**	300.00	98.23	4.71	91.54	9.32	103.46	12.06
	3.00	100.29	1.81	87.07	9.21	103.12	8.24
	600.00	97.45	1.87	114.62	2.85	100.76	2.01
**33**	300.00	103.97	3.50	118.08	1.18	101.38	1.20
	3.00	99.94	2.56	115.46	2.89	100.65	1.71
	1000.00	105.41	3.91	95.54	6.06	100.07	4.70
**34**	500.00	98.99	2.39	107.92	7.58	100.08	10.06
	5.00	108.74	10.12	106.08	9.52	99.03	0.91
	1000.00	103.35	2.84	117.44	2.36	100.45	8.42
**35**	500.00	103.86	4.64	117.00	0.94	100.34	8.11
	25.00	98.89	6.24	100.19	12.77	92.67	1.26
	600.00	102.73	5.56	114.13	3.62	101.12	3.70
**36**	300.00	104.23	1.30	117.55	2.18	101.08	0.92
	15.00	101.15	3.44	105.77	11.38	101.28	11.71
	1000.00	108.73	0.88	106.20	3.88	100.80	0.93
**37**	500.00	105.04	4.70	108.81	9.87	96.89	5.56
	5.00	96.21	2.54	91.46	9.31	100.06	11.33
	600.00	96.72	3.41	106.64	5.76	100.44	4.20
**38**	300.00	106.77	4.98	114.37	3.43	101.03	11.51
	3.00	103.10	4.56	101.36	8.89	111.34	10.02
	1000.00	101.57	4.64	110.06	6.37	100.49	2.45
**39**	250.00	93.91	5.47	101.79	3.66	102.12	7.01
	5.00	97.30	2.97	110.52	5.48	103.32	10.20
	1000.00	117.16	0.99	113.48	3.84	100.50	3.42
**40**	500.00	101.74	3.19	108.53	6.11	100.34	2.84
	5.00	102.12	1.02	94.27	3.56	107.71	6.55
	1000.00	92.63	5.79	95.63	12.20	100.43	1.50
**41**	500.00	97.88	4.00	107.80	11.92	101.72	2.59
	5.00	99.88	2.73	100.16	14.74	101.60	5.82

	600.00	100.68	0.80	117.06	0.69	100.32	2.03
**42**	300.00	104.34	2.30	118.55	0.72	101.18	0.64
	3.00	102.93	1.26	117.01	1.25	101.29	1.01

4.5. Stability

The freeze and thaw stability, short-term and long-term stabilities were determined at high and low concentrations after 24 h storage in the sample tray, after one, and twenty-day storage at −80 °C, respectively. All the analytes showed variations between -17.26% and 17.44% (Table S5). The results indicated that these analytes in rats plasma were all stable for twenty-day storage at −80 °C, 24 h in the auto-sampler (4 °C) and three freeze–thaw cycles.

TABLE S5. Stability of the analytes (n = 5).

Analyte	Calc. Con. (ng/ml)	Freeze and Thaw		Short-Term		Long-Term	
		Remain (%)	RSD (%)	Remain (%)	RSD (%)	Remain (%)	RSD (%)
**1**	600.00	96.48	12.70	94.29	8.89	100.56	7.31
	3.00	90.89	14.39	85.41	5.88	94.78	9.69
**2**	1000.00	116.88	3.43	116.92	4.73	116.20	3.43
	5.00	103.13	11.34	102.43	11.90	105.74	2.01
**3**	600.00	86.60	12.92	82.74	2.26	83.79	3.64
	3.00	93.14	11.45	88.50	8.53	91.87	8.88
**4**	1000.00	117.11	1.03	116.88	0.82	115.39	4.94
	5.00	110.01	11.29	104.30	14.25	112.61	5.32
**5**	600.00	112.63	2.22	113.20	2.36	114.38	4.69
	3.00	93.08	11.93	87.94	5.85	97.98	7.69
**6**	500.00	112.20	3.57	108.63	7.98	114.86	2.44
	5.00	93.92	12.94	91.30	14.67	100.38	14.17
**7**	600.00	109.74	3.43	110.81	4.73	110.57	3.43
	3.00	104.01	11.34	106.75	11.90	111.53	2.01
**8**	600.00	97.73	12.26	93.76	8.65	97.99	5.43
	3.00	93.45	4.06	91.55	6.85	95.80	7.96
**9**	600.00	91.18	9.44	90.17	7.72	96.64	7.82
	3.00	97.51	6.17	97.06	6.01	93.06	9.94
**10**	1000.00	108.56	10.86	107.95	10.67	116.28	3.38
	10.00	98.56	12.97	93.87	10.70	103.92	7.82
**11**	1000.00	116.73	1.71	115.13	2.56	114.23	5.88
	10.00	92.99	10.71	96.20	10.58	99.85	5.46
**12**	600.00	100.61	9.02	99.56	7.15	101.13	6.23

	3.00	96.35	8.94	98.54	11.35	98.29	8.04
**13**	600.00	116.15	1.30	115.49	2.67	112.33	5.30
	3.00	98.51	8.07	96.41	8.74	97.37	7.84
**14**	1000.00	116.83	1.68	116.37	1.22	114.01	5.61
	5.00	95.81	10.39	94.45	10.78	96.45	9.60
**15**	600.00	114.70	3.33	115.28	3.53	113.11	3.53
	3.00	104.14	8.84	107.24	7.37	108.46	7.67
**16**	600.00	116.14	1.32	115.40	1.60	111.22	4.03
	3.00	111.97	4.72	112.20	4.76	111.86	5.99
**17**	600.00	116.18	1.44	113.62	4.75	110.08	5.65
	3.00	102.88	4.84	99.96	5.63	96.98	7.81
**18**	600.00	110.60	6.77	107.84	11.70	115.67	2.54
	3.00	96.34	9.20	97.90	5.81	102.14	7.74
**19**	1000.00	100.88	9.78	93.74	9.01	105.99	8.55
	50.00	99.08	14.05	96.49	12.76	87.84	7.71
**20**	500.00	99.11	13.13	95.57	10.92	111.62	5.21
	25.00	105.94	11.24	103.90	14.60	107.05	12.19
**21**	600.00	98.67	8.82	94.85	1.02	96.16	3.30
	3.00	108.37	12.69	109.65	11.96	116.03	2.11
**22**	600.00	94.80	13.17	89.38	6.30	85.00	5.06
	3.00	106.98	10.31	101.11	12.68	107.31	6.06
**23**	1000.00	99.74	11.14	85.12	7.41	103.31	10.39
	5.00	111.71	6.13	110.07	6.21	106.01	11.60
**24**	600.00	115.49	4.29	115.49	4.29	99.82	8.75
	3.00	108.24	2.04	107.71	2.35	108.49	2.43
**25**	1000.00	99.31	10.42	99.02	9.81	95.71	4.04
	5.00	96.47	12.06	92.91	7.03	91.33	4.69
**26**	1000.00	94.84	6.91	94.64	7.15	106.05	6.79
	5.00	100.34	6.64	100.89	7.14	102.71	3.06
**27**	1000.00	97.63	6.74	95.12	8.06	108.10	6.83
	5.00	92.95	12.55	88.87	5.11	93.06	4.53
**28**	600.00	94.41	9.81	91.90	3.88	89.49	2.67
	3.00	87.96	6.07	88.05	6.11	92.13	2.58
**29**	600.00	107.61	4.02	108.20	5.27	105.12	1.91
	3.00	98.75	14.57	96.05	11.83	92.46	5.48
**30**	600.00	101.87	6.76	98.17	3.59	91.95	9.14
	3.00	101.50	7.43	99.97	7.70	106.01	5.28
**31**	1000.00	109.13	6.35	110.91	6.98	115.15	3.94
	5.00	94.36	6.29	97.45	9.46	99.01	4.38
**32**	600.00	95.09	10.51	91.80	4.20	93.53	1.66
	3.00	91.82	15.15	87.54	8.54	91.25	8.99
**33**	600.00	111.70	3.79	112.85	4.37	110.98	2.80
	3.00	114.58	2.70	115.54	2.47	116.79	2.27
**34**	1000.00	96.52	6.81	97.91	6.44	107.23	8.74

	5.00	107.54	9.34	103.81	9.42	96.44	11.92
**35**	1000.00	117.73	2.19	117.44	2.12	115.96	3.73
	25.00	101.15	4.24	101.35	12.03	105.45	6.68
**36**	600.00	113.13	3.65	112.15	2.67	107.82	6.33
	15.00	103.43	12.09	102.53	11.11	109.85	6.22
**37**	1000.00	109.87	6.74	108.28	5.18	111.53	2.95
	5.00	95.68	13.58	86.54	5.37	104.84	9.68
**38**	600.00	107.48	7.19	107.54	7.25	106.05	4.77
	3.00	101.68	10.55	101.67	10.54	96.69	8.38
**39**	1000.00	108.35	8.37	108.07	8.06	108.24	4.12
	5.00	108.04	6.62	108.15	6.53	106.22	5.24
**40**	1000.00	113.20	4.21	112.84	3.75	104.49	10.13
	5.00	97.58	5.89	100.32	7.96	96.72	3.50
**41**	1000.00	100.94	12.11	96.94	9.68	97.40	10.87
	5.00	93.59	11.42	92.73	11.86	95.96	9.96
**42**	600.00	117.32	0.82	116.54	1.26	111.62	5.89
	3.00	116.80	1.21	117.00	1.13	116.07	2.37

5. Qualitative and quantitative analysis of Gegen-Qinlian Decoction

An Agilent 1260 series HPLC instrument (Agilent, Waldbronn, Germany) was used to obtain the chromatogram of Gegen Qinlian Decoction. Samples were separated on an XTerra MS C18 column (5 μm, ID 4.6 × 250 mm) (Waters, MA, USA). The mobile phase consisted of acetonitrile (A) and water containing 0.1% (*v*/*v*) formic acid (B). A gradient program was used as follows: 0 min, 5% A; 20 min, 15% A; 24 min, 19% A; 32 min, 27% A; 40 min, 50% A; 44 min, 60% A; 52 min, 80% A; 53 min, 100% A; 55 min, 100% A. The flow rate was 0.8 mL/min and the column temperature was 50 °C. The detector wavelength was 270 nm. For quantitative analysis of compounds **35**, **41**, and **42**, the decoction was diluted for 100 fold using 67% methanol, and analyzed using the LC/MS/MS method described in the manuscript.

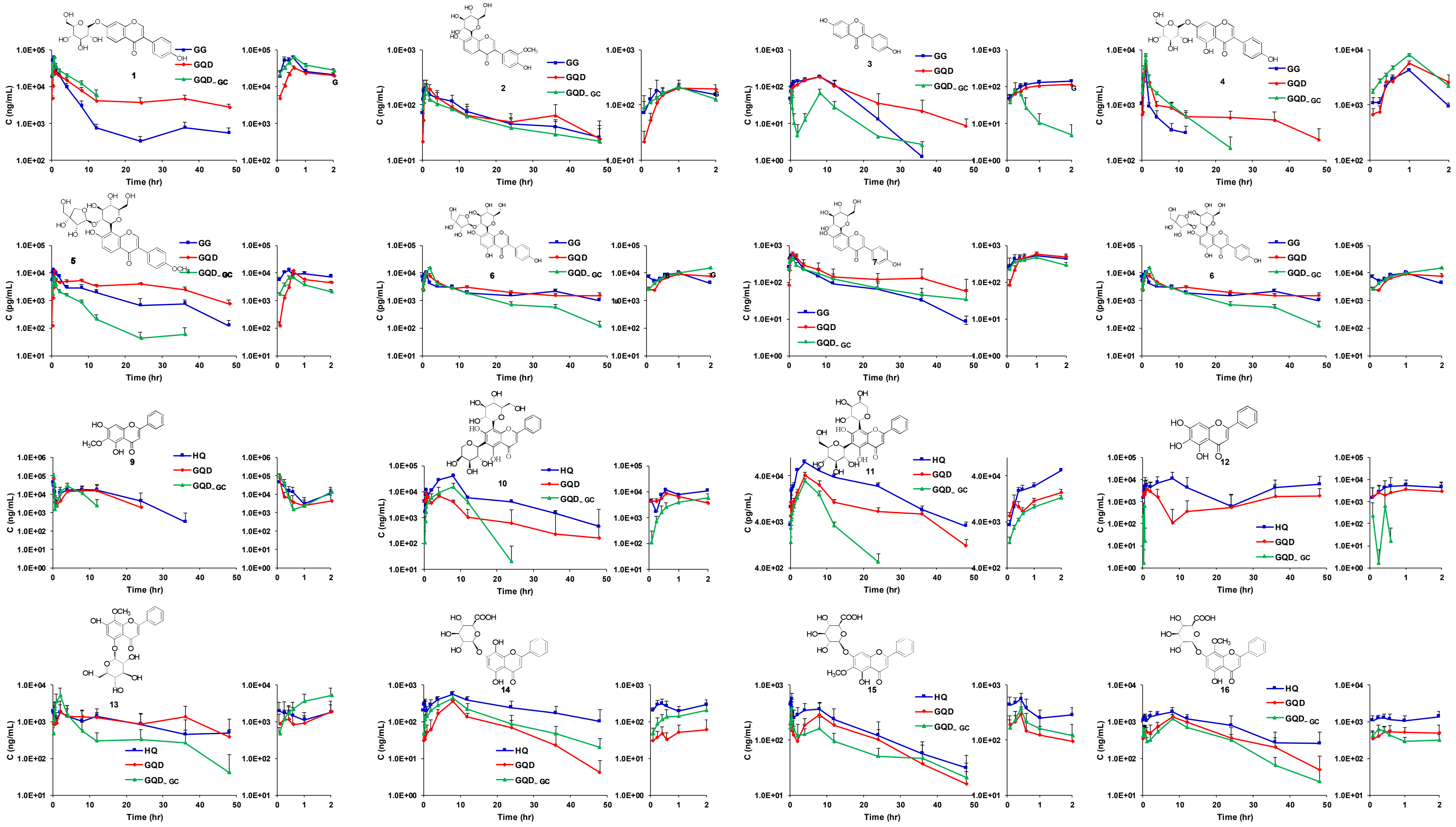
1
2
3
4
5
6
7
6
9
10
11
12
13
14
15
16
GG
GQD
GQD- GC
HQ
GQD
GQD- GC
C (ng/mL)
C (pg/mL)
Time (hr)

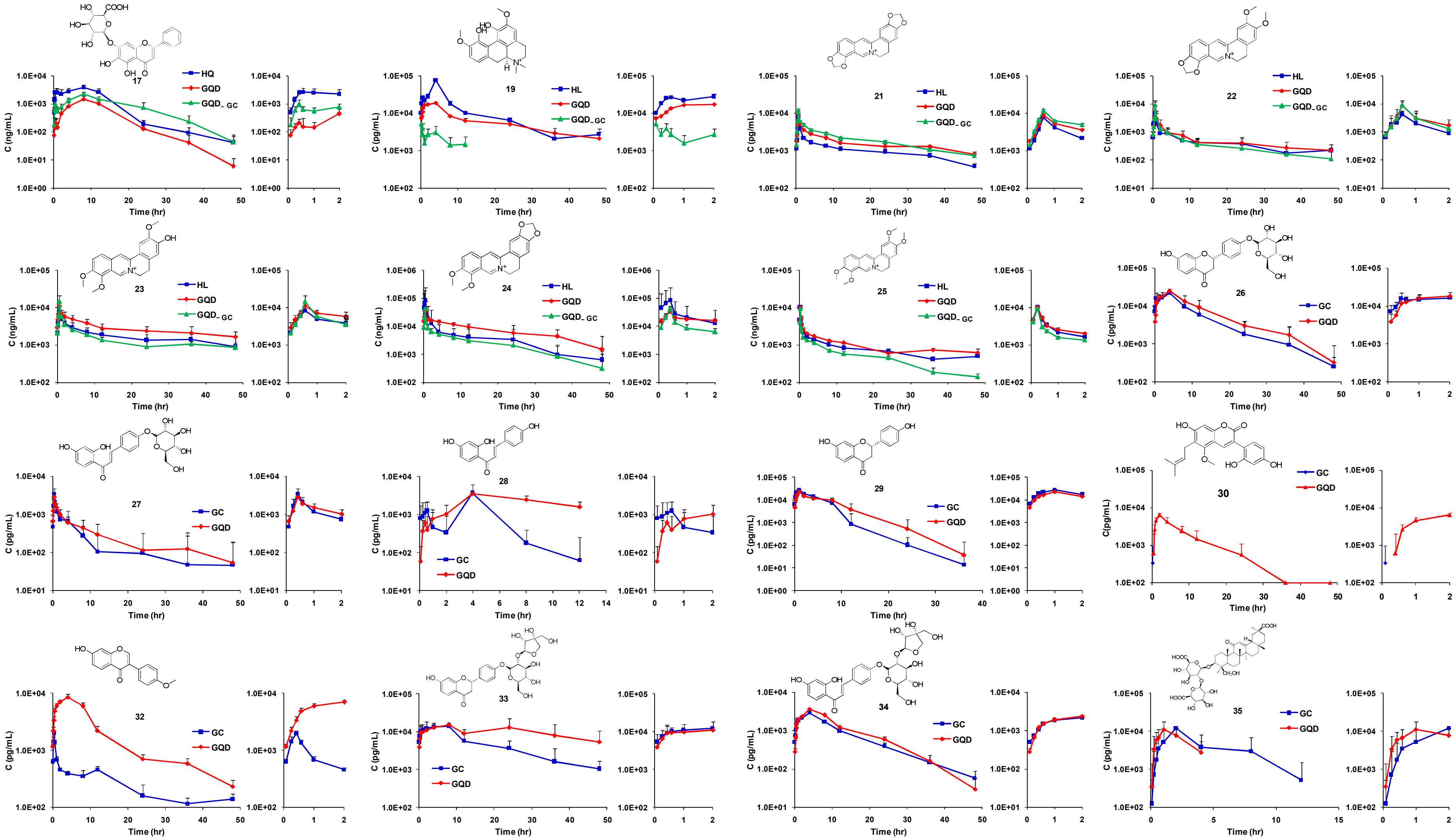
17
HQ
GQD
GQD_GC
C (ng/mL)
Time (hr)
19
HL
GQD
GQD_GC
C (pg/mL)
21
22
23
24
25
26
27
28
29
30
32
33
34
35
GC
GQD
C(pg/mL)

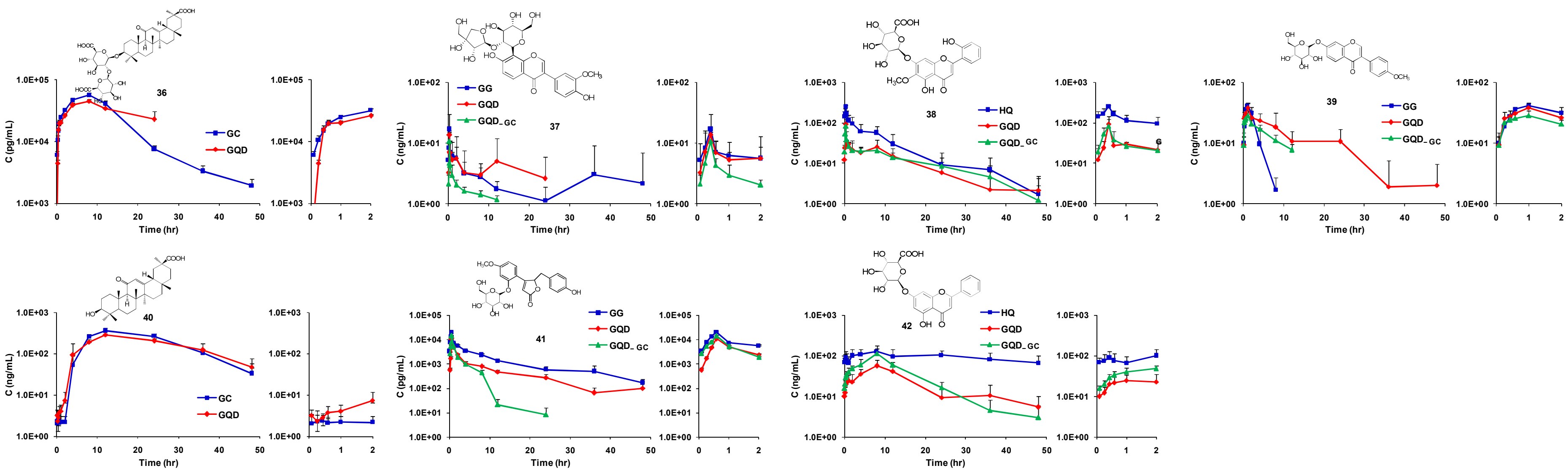

Fig. S2. Time-plasma concentration curves of 42 markers in the component herb, GQD, and GQD$_{-GC}$ groups.

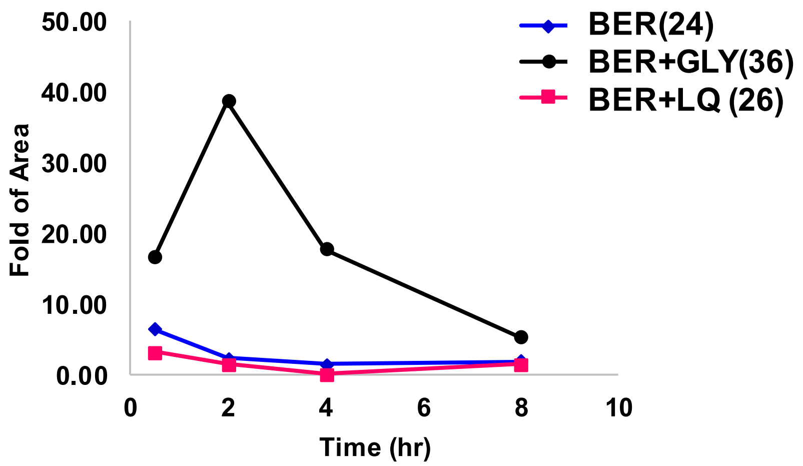

Fig. S3. A preliminary study on the impact of glycyrrhizic acid (GLY, **36**, 100 mg/kg) and liquiritin (LQ, **26**, 50 mg/kg) on the pharmacokinetics of berberine (BER, **24**, 100 mg/kg) in rats. *n*=1 for each group.

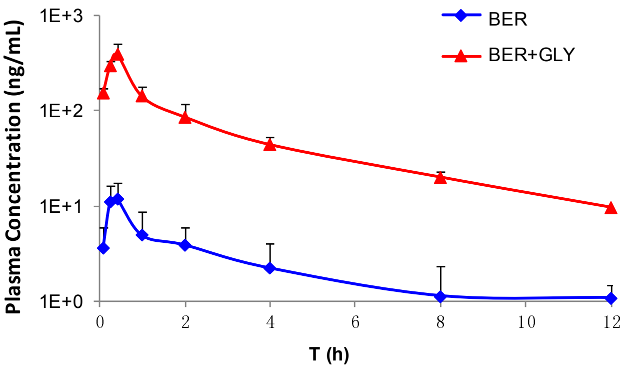

Fig. S4. Time-plasma concentration curves of berberine in rats orally administered with berberine (BER, *n*=8), or berberine and glycyrrhizic acid (GLY, *n*=8).

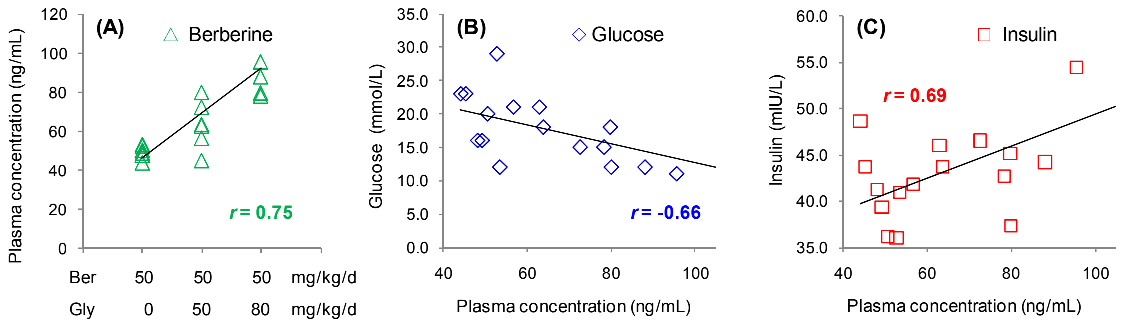

Fig. S5. Correlations of dosage, berberine plasma concentration, and anti-diabetic effects of BER+GLY combinations in db/db mice. (A) Plasma concentrations of berberine (50 mg/kg) co-administered with glycyrrhizic acid (0, 50, 80 mg/kg). (B) PK-PD correlation of berberine concentration with serum glucose level (mmol/L). (C) PK-PD correlation of berberine concentration with serum creatinine level (mIU/L). *r* indicates Pearson correlation coefficient.
